# Two approaches reveal a new paradigm of ‘switchable or genetics-influenced allele-specific DNA methylation’ with potential in human disease

**DOI:** 10.1038/celldisc.2017.38

**Published:** 2017-11-14

**Authors:** Suzanne N Martos, Teng Li, Ramon Bossardi Ramos, Dan Lou, Hongzheng Dai, Jin-Chong Xu, Ganglong Gao, Yang Gao, Qinglu Wang, Cheng An, Xueli Zhang, Yankai Jia, Valina L Dawson, Ted M Dawson, Hongkai Ji, Zhibin Wang

**Affiliations:** 1 Laboratory of Human Environmental Epigenomes, Department of Environmental Health & Engineering, Bloomberg School of Public Health, Johns Hopkins University, Baltimore, MD, USA; 2 Neuroregeneration and Stem Cell Programs, Institute for Cell Engineering, Johns Hopkins University, Baltimore, MD, USA; 3 Department of Neurology, Johns Hopkins University, Baltimore, MD, USA; 4 Fenxian Central Hospital, Shanghai, China; 5 GENEWIZ Suzhou, Suzhou, China; 6 Department of Solomon H. Snyder Department of Neuroscience, Johns Hopkins University, Baltimore, MD, USA; 7 Department of Physiology, School of Medicine, Johns Hopkins University, Baltimore, MD, USA; 8 Department of Pharmacology and Molecular Sciences, Baltimore, MD, USA; 9 Department of Biostatistics, Bloomberg School of Public Health, Johns Hopkins University, Baltimore, MD, USA; 10 School of Life Sciences, Hubei University, Wuhan, China; 11 The Sidney Kimmel Comprehensive Cancer Center and Department of Oncology; School of Medicine, Johns Hopkins University, Baltimore, MD, USA

**Keywords:** allele-specific methylation (ASM), genomic imprinting, NORED, MethylMosaic, autosomal chromosome inactivation (ACI)

## Abstract

Imprinted genes are vulnerable to environmental influences during early embryonic development, thereby contributing to the onset of disease in adulthood. Monoallelic methylation at several germline imprints has been reported as DNMT1-dependent. However, which of these two epigenetic attributes, DNMT1-dependence or allelic methylation, renders imprinted genes susceptible to environmental stressors has not been determined. Herein, we developed a new approach, referred to as NORED, to identify 2468 DNMT1-dependent DNA methylation patterns in the mouse genome. We further developed an algorithm based on a genetic variation-independent approach (referred to as MethylMosaic) to detect 2487 regions with bimodal methylation patterns. Two approaches identified 207 regions, including known imprinted germline allele-specific methylation patterns (ASMs), that were both NORED and MethylMosaic regions. Examination of methylation in four independent mouse embryonic stem cell lines shows that two regions identified by both NORED and MethylMosaic (*Hcn2* and *Park7*) did not display parent-of-origin-dependent allelic methylation. In these four F1 hybrid cell lines, genetic variation in Cast allele at *Hcn2* locus introduces a transcription factor binding site for MTF-1 that may predispose Cast allelic hypomethylation in a reciprocal cross with either C57 or 129 strains. In contrast, each allele of *Hcn2* ASM in J1 inbred cell line and *Park7* ASM in four F1 hybrid cell lines seems to exhibit similar propensity to be either hypo- or hypermethylated, suggesting a ‘random, switchable’ ASM. Together with published results, our data on ASMs prompted us to propose a hypothesis of regional ‘autosomal chromosome inactivation (ACI)’ that may control a subset of autosomal genes. Therefore, our results open a new avenue to understand monoallelic methylation and provide a rich resource of candidate genes to examine in environmental and nutritional exposure models.

## Introduction

Environmental factors impact human health [1–3]. For example, a high fat diet contributes to the pathogenesis of obesity and type 2 diabetes [4, 5], and low nutrition during early embryonic development may link to cardiovascular diseases in later life; [6]. and exposure to endocrine disrupting chemicals such as Bisphenol A increases the susceptibility to diseases including neurological disorders [7–9], heart diseases [10, 11]. and cancers [12–22]. These factors contribute to the disease pathogenesis, largely via epigenetic mechanisms [2, 3, 5, 23, 24]. Altered epigenetic patterns have been involved in numerous human diseases. A notable example is that cancer cells are characterized by genome-wide hypomethylation and region-specific hypermethylation. Additional examples include abnormal hypomethylation of genes involved in autoimmune diseases such as systemic lupus erythematosus [25]. For mechanistic insights of such alteration in diseases, investigators have been focusing on exposure-induced changes in expression or function of DNA methyltransferases (DNMTs) [24]. Many publications report altered expression of DNMT1, the maintenance enzyme for transmitting DNA methylation patterns in generations of somatic cells [24]. However, DNMT-dependent alterations of DNA methylation in diseases remain to be determined. A systematic examination of DNMT-dependent methylation regions via global profiling of DNMT-deficient cells will significantly improve our understanding of such alterations in diseases.

Imprinted genes are associated with monoallelic methylation and monoallelic gene expression. As such, they are considered particularly vulnerable to environmental exposure [26–28]. Monoallelic expression of imprinted genes is controlled by germline allele-specific methylation (ASM; traditionally called as differentially methylated region (DMR)). For these germline ASMs (gASMs), CpGs at the imprinting control regions of one parental allele are methylated, whereas these of another allele are unmethylated. When examined by bisulfite Sanger sequencing, half of PCR clones in those regions are hypermethyated and half are hypomethylated, thereby showing bimodal methylation patterns. Importantly, these gASMs/DMRs are considered stable during the cycles of global demethylation and remethylation during early embryo development [29]. Once these ASMs have been altered due to exposure in sperms and/or oocytes, such alteration could be carried as ‘epigenetic memories’ to somatic cells. Numerous studies focus on the alteration of these ASM-controlled imprinted genes for understanding of ‘developmental origin of adult disease’ and ‘transgenerational epigenetic inheritance’ of disease [26, 30]. However, most current studies use a candidate approach that targets several imprinted ASMs (or imprinted genes), particularly *Igf2* [26]. For example, in a widely cited study, the methylation level of several CpG sites of *Igf2* ASM from Dutch famine patients was shown to be maintained after decades [31]. Limited work has been done to systematically examine all gASMs, including their exact sizes and the possibility of unknown gASMs. Such a genome-wide examination will be helpful for understanding the pathogenesis and diagnosis of human diseases/syndromes.

In addition to the above-mentioned parent-of-origin-specific monoallelic expression, the mammalian genome has a surprisingly large number of genes showing random monoallelic expression (RME). [32–36]. Although genetic variants can affect expression, such monoallelic expression is unexpected, as the conventional notion is that non-imprinted genes on autosomal chromosomes should be either bialellically expressed or biallelically repressed. Monoallelic gene expression was previously thought to occur only at imprinted loci or genes from X chromosomes for which one chromosome (paternal or maternal) is randomly inactivated in females [37]. However, recent studies found that the monoallelic expression of non-imprinted, autosomal genes does not seem to be a sporadic phenomenon, but a conserved feature in both the mouse genome [34–36]. and the human genome [32, 33]. While RMEs seem to occur frequently, the underlying mechanism remains elusive. Two recent investigations suggest epigenetic mechanism cannot account for RME [35, 36]. In addition, the role of monoallelically expressed genes in both development and diseases such as cancers [38]. needs urgent investigation (see the significance as exemplified by this work later).

Monoallelic methylation (that is, imprinted ASM) or expression has been provided as a rationale to explain why imprinted genes are susceptible to nutritional and environmental influences. With similar monoallelic methylation/expression, it is reasonable to expect that other ASMs or RMEs would demonstrate similar vulnerability. However, the molecular explanation for these expectations remains to be fully explored. Early studies have identified several gASMs as highly DNMT1-dependent in preimplantation embryos [39]. Specifically, global methylation substantially recovered in ‘rescued’ DNMT1-deficient embryonic stems cells (ESCs), whereas the methylation at several gASMs, which had been abolished in DNMT1-deficient ESCs, was not restored in ‘rescued’ DNMT1-deficient ESCs [39]. Notably, while DNMT1-deficiency is embryonic lethal [40], ‘rescued’ DNMT1-deficient ESCs could contribute to viable adult chimeras [39]. In contrast, overexpression of DNMT1, which resulted *de novo* methylation at the unmethylated allele of *Igf2*, was embryonic lethal [41]. Taken together, these early studies suggest that while DNMT1 is required to maintain imprinted ASMs, hypermethylation is not compatible with embryonic viability, whereas hypomethylation of the methylated allele for some gASMs may be tolerated. If the finding of DNMT1-dependent DNA methylation loss in preimplantation embryos can be can be extended to additional imprinted gASMs, then this apparent vulnerability could provide an alternate explanation for why gASMs of imprinted genes are considered especially susceptible to environmental influences. Yet, this raises the question as to which of two epigenetic attributes, DNMT1-dependence or allelic methylation, renders gASMs susceptible to environmental stressors. Furthermore, it raises the question as to whether additional genomic regions display non-restorable DNMT1-dependent methylation loss.

To provide mechanistic insights for questions above, herein we investigate regions throughout the genome that exhibit DNMT1-dependence and/or have bimodal methylation patterns. We begin with examining DNA methylation patterns associated with genomic imprinting, which is complementary to our previous investigation of methylation patterns associated with gene transcription and genome stability (that is, suppression of transposable elements) [42]. On the basis of the loss of gASMs/DMRs in DNMT1 knockout (1KO) ESCs and failure to restore the loss in 1KO cells with exogenous expression of DNMT1 cDNA, we developed a new approach, ‘non-rescued DMR (NORED)’, identifying genomic regions dependent on DNMT1. Among these regions, many are bona fide imprinted gASMs with the expected bimodal methylation patterns, as unveiled by our ‘MethylMosaic’ analyses. In addition to the known imprinted gASMs, MethylMosaic analyses also identify genomic regions showing bimodal methylation patterns. We next generate four independent mouse ESC lines from hybrid mice and demonstrate that some NORED regions with bimodal methylation patterns show allelic methylation, but in a parent-of-origin-independent manner. Intriguingly, genetic differences at *Hcn2/Polrmt* locus predisposed Cast allele to be hypomethylated in cross with either 129 or C57, whereas genetic differences at *Park7* locus did not. Their shared features (for example, allelic hyper-/hypomethylation) with X chromosome inactivation (XCI) raise the possibility that many genes on autosomal chromosomes are controlled by an XCI-like mechanism of regional ‘autosomal chromosome inactivation (ACI).’

## Results

### All gASMs are lost in DNMT1-deficient ESCs, whereas specific loci exhibit resistance to methylation loss in DNMT3a/3b-deficient ESCs

To determine DNA methylation patterns’ impacts on gene transcription, genome stability, and genomic imprinting, we have characterized the base resolution DNA methylomes of wild type (WT, J1 ESC line) and DNMT-deficient ESCs, including the loss of maintenance DNMT1 (DNMT1^−/−^, 1KO), of two *de novo* DNMT3a/3b (DNMT3a^−/−^/3b^−/−^, DKO), and of all three (DNMT1^−/−^/3a^−/−^/3b^−/−^, TKO; Supplementary Figure S1A, B, D and E). Genome-wide, average methylation was 0.727, 0.176, 0.157, and 0.006 in WT, 1KO, DKO, and TKO, respectively. Previously, we reported on the distinct roles of DNMT1-dependent and -independent methylation patterns in suppression of transposable elements and the complete hypomethylation on induction of only small number of genes in the mouse genome [42]. Herein we focused our study on DNA methylation patterns at imprinted loci.

**Figure 1:**
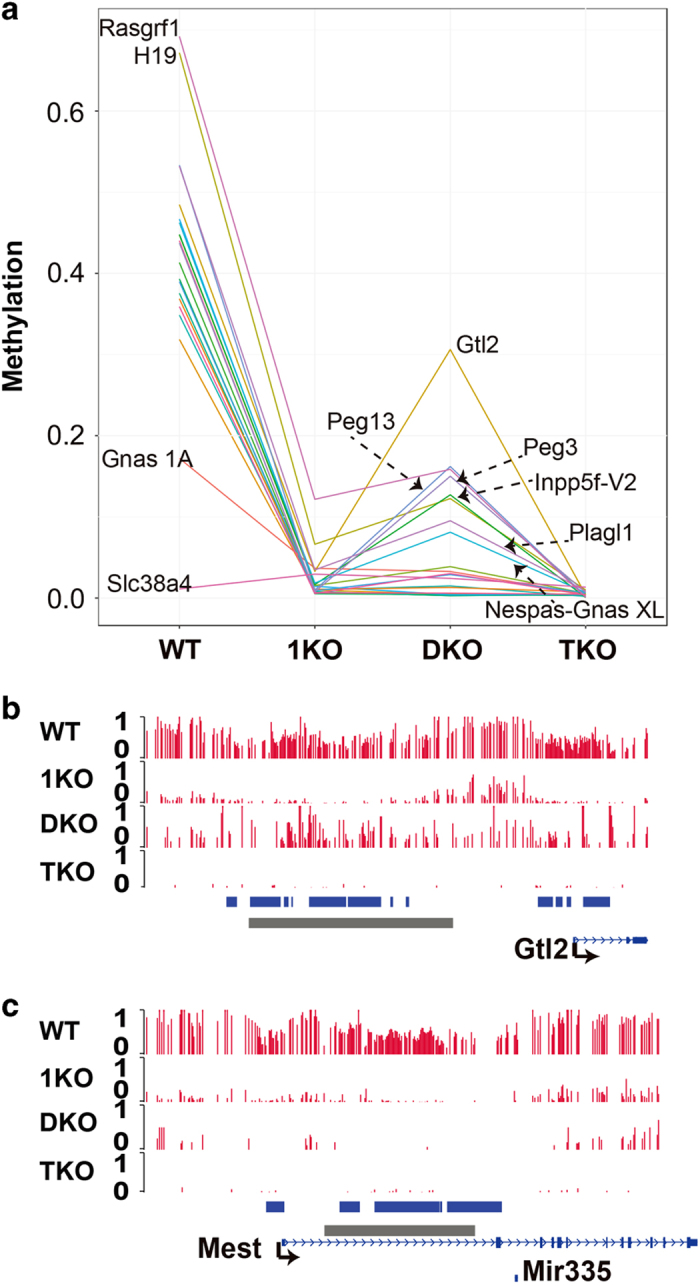
Germline ASMs (gASMs) are lost in DNMT1-deficient ESCs, whereas specific loci exhibit resistance to methylation loss in DNMT3a/3b-deficient ESCs. (**a**) Genome-wide profiling of gASM methylation level in WT and DNMT mutant mouse ESCs. Each line represents a single gASM. Data shown for 21 well-characterized gASMs. Eleven gASMs (unlabeled) have average methylation levels consistent with the expectation of one methylated allele and one unmethylated allele in WT, experience near complete loss of methylation in 1KO and DKO, and complete methylation loss in TKO. *Rasgrf1* and *H19* gASMs have methylation levels somewhat higher than the expectation of one methylated allele and one unmethylated allele in WT, retain partial methylation in 1KO and DKO, and experience complete loss of methylation in TKO. Six gASMs (*Peg13, Gtl2, Peg3, Inpp5f-*V2, *Plagl1* and *Nespas-Gnas* XL) have methylation levels consistent with the expectation of one methylated allele and one unmethylated allele in WT, experience near complete loss of methylation in 1KO, retain partial methylation in in DKO and experience complete loss of methylation in TKO. *Gnas* 1A has partial methylation in WT and experiences near complete loss of methylation in WT, 1KO and TKO. *Slc38a4* has low methylation in in WT, 1KO, DKO, and TKO. Genome-wide, average CpG methylation on a zero to one scale was 0.727, 0.176, 0.157 and 0.006 in WT, 1KO, DKO and TKO, respectively. See Supplementary Table S1 for locus-specific methylation levels. (**b**) *Gtl2* gASM is abolished in 1KO cells. In contrast, DKO cells retain partial methylation patterns. (**c**) Methylation at *Mest* locus is completely abolished in both 1KO and DKO cells. (**b** and **c**) Blue bars indicate NORED regions. Gray bars indicate gASMs.

DNA methylation is essential for genomic imprinting [43, 44]. To determine the extent to which gASMs are dependent on maintenance DNMT1 or *de novo* DNMT3a/b for methylation maintenance, we compared the methylation levels of 21 well-characterized gASMs found in common between two sources [45, 46]. among WT, 1KO, DKO, and TKO ESCs (Figure 1a and Supplementary Table S1). Except for 1KO versus DKO, pairwise comparisons were significant (Bonferroni corrected *P*<0.05). Methylation levels of imprinted gASMs were significantly reduced in 1KO, DKO, and TKO compared to WT (Bonferroni corrected *P*=1.1×10^−5^; Figure 1a and Supplementary Table S1). Compared to TKO, imprinted gASMs had significantly higher methylation in both 1KO (Bonferroni-corrected *P*=5.7×10^−6^) and DKO (Bonferroni-corrected *P*=6.3×10^−4^; Figure 1a and Supplementary Table S1). However, methylation of imprinted gASMs did not differ significantly between 1KO and DKO (Bonferroni-corrected *P*=0.085; Figure 1a and Supplementary Table S1). Therefore, in general, methylation maintenance at known gASMs is dependent on activity from DNMT1 and DNMT3a/3b in ESCs.

Targeted studies at a few gASMs have reported ‘near complete’ abolishment of allelic methylation upon the loss of DNMT1 in preimplantation embryos [41, 47]. To compare these reported results with our findings, we examined the methylation level at individual loci. Although ‘near complete’ methylation loss was not quantitatively defined [47], loci presented had at most 5% methylation. From our data of 1KO ESCs, only two gASMs had >5% methylation: *Rasgrf1* (12.2%) and *H19* (6.6%; Figure 1a and Supplementary Table S1). Methylation at other imprinted loci, including *Gtl2* (*Meg3*) and *Mest*, is indeed abolished (Figure 1a–c and Supplementary Table S1). This is consistent with reports that DNMT1 is necessary for the maintenance of imprinted gASMs [41, 47].

Contrary to the indispensable role of maintenance DNMT1 above, *de novo* activities of DNMT3a/3b were reported to be dispensable for the maintenance of two paternally methylated DMRs, *H19* and *Gtl2*, as neither DMR was affected. For another paternally methylated locus, *Rasgrf1*, the reduced methylation was attributed to an unusual repeat structure at this region [47]. However, using our base-resolution methylome data (which covers the entire gASM instead of a portion as in [47].), we found that methylation at *H19* and *Rasgrf1* was substantially reduced, whereas *Gtl2* only decreased by 0.18 (Figure 1a and b and Supplementary Table S1). In total, eight gASMs (which represent both maternally and paternally methylated loci) had >5% methylation in DKO (DNMT3a/3b-deficient and DNMT1 intact) ESCs: *Gtl2* (30.6%), *Peg13*/*Trappc9* (16.2%), *Rasgrf1* (15.9%), *Peg3* (15.0%), *Inpp5f-v2* (12.7%), *H19* (12.3%), *Plagl1* (9.5%) and *Nespas-GnasXL* (8.1%; Supplementary Table S1). Strikingly, *Mest* and remaining gASMs were completely abolished (Figure 1a and c and Supplementary Table S1), suggesting an indispensable role of DNMT3a/3b in the maintenance of certain gASMs.

Altogether, we conclude that DNMT1 is necessary, but not sufficient to maintain methylation at gASMs and specific loci exhibit partial resistance to methylation loss in the absence of DNMT3a/3b. These facts reveal the previously unappreciated coordination between *de novo* and maintenance activities to maintain methylation patterns, which was also demonstrated by us previously [42]. These facts are different from the traditional view/model of ‘DNMT3a/3b for initiating methylation and DNMT1 for maintaining afterward.’

### Loss of methylation was not rescued at gASMs

**Figure 2:**
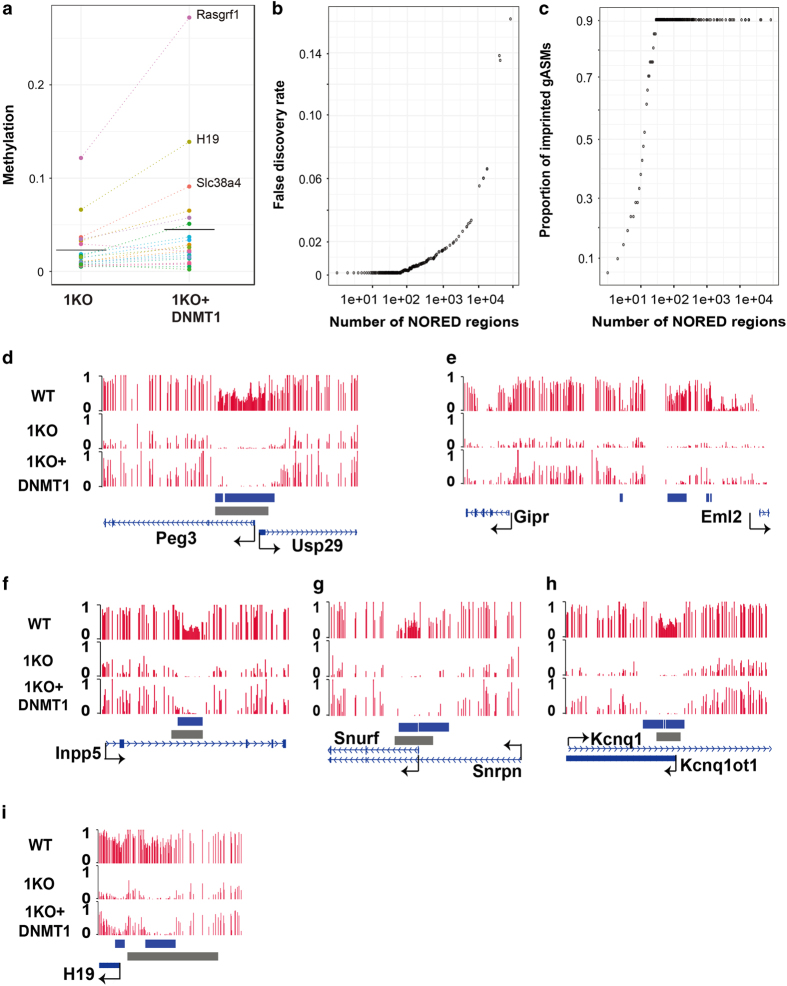
Loss of methylation was not rescued at gASMs and other specific loci. (**a**) Profiling of gASM methylation level in 1KO and DNMT1-rescued 1KO (r1KO) ESCs. For most gASMs, low methylation in 1KO is not substantially increased by exogenous expression of DNMT1 cDNA in r1KO ESCs. Genome-wide, average CpG methylation on a zero to one scale was 0.369 for r1KO cells. See Supplementary Table S1 for locus-specific details. (**b**) False discovery rate (FDR, *y* axis) of NORED as a function of rank (*x* axis) for NORED regions. (**c**) Proportion of well-established gASMs (*y* axis) identified by NORED as a function of rank (*x* axis) for NORED regions. Nineteen gASMs rank among top 29 NORED regions. (**d**) Deficiency of methylated CpG sites (red bars) in 1KO and DNMT1-rescued 1KO cells can be used to identify and demarcate the known *Peg3* DMR. (**e**) NORED identifies *Gipr*/*Eml2* locus (imprinted status unknown). (**f**) NORED identifies, gASM in *Inpp5f* locus. (**g**) NORED improves the demarcation of gASMs for Prader-Willi and Angelman syndromes (*Snrpn*/*Snurf*). (**h**) NORED demarcates region larger than known gASM at *Kcnq1/Kcnqot1* locus. (**i**) NORED successfully identifies gASM and somatic ASM at near *H19*. (**d**–**i**) Blue bars indicate NORED regions. Gray bars indicate location of previously established gASMs.

Recognizing that important gASMs require DNMT1 for maintenance of DNA methylation, we wondered whether loss of methylation at these loci could be restored once it had been abolished. Literature search indicated that exogenous expression of DNMT1 cDNA did not restore methylation at a few gASMs [41]. To extend this finding to other well-characterized gASMs, we expressed DNMT1 in 1KO cells to characterize the base resolution DNA methylome of ‘rescued 1KO’ ESCs (DNMT1^−/−^+DNMT1, r1KO; Supplementary Figure S1C). Global average methylation in r1KO (0.369) increased to approximately 50.8% of WT levels, whereas average 1KO levels were 24.1% that of WT (Supplementary Figure S1F). Therefore, we consider global methylation to be substantially restored in r1KO ESCs. In contrast, average methylation for gASMs decreased to 0.023 (5.4% of WT levels at these loci) in 1KO and recovered to only 0.045 (10.8% of WT levels at these loci) in r1KO (Figure 2a and Supplementary Table S1).

### NORED: genome-wide detection of DNMT1-dependent methylation that is not recovered once abolished

We took advantage of the previous observation to develop NORED, a new method to systematically identify genomic regions with non-rescued DMR. These NORED regions must have sufficient methylation in WT, near complete loss of methylation in 1KO, and minimum recovery of methylation in r1KO. We performed a receiver operating characteristic (ROC)-like analysis using a permutation-based approach to estimate the false positives at various methylation cutoffs for WT, 1KO, and r1KO ESCs (Supplementary Figure S2A). At a false positive rate (FPR) of 0.01, 70% of CpGs within gASMs had at least 25% methylation in WT, at most 5% methylation in 1KO, and at most 12.5% methylation in r1KO (Supplementary Figure S2A). By contrast, genome-wide only 3.3% of CpG sites met these criteria. We then clustered individual CpG sites into regions and ranked the resulting NORED regions based on the number of CpG sites included and the proportion of consecutive CpG sites that met the criteria (see Materials and Methods). To determine the false discovery rate (FDR) for NORED, we applied the clustering and scoring algorithms to permuted data and estimated the average FDR based on twenty permutations.

NORED analyses identified 2468 regions at FDR=0.02 (Figure 2b, Supplementary Figure S2B, Supplementary Table S2). The highest ranked 207 regions (FDR<5×10^−3^) are presented in Table 1. Only two gASMs, *Slc38a4* and *Gnas 1A*, were not identified by NORED because neither of these regions had sufficient methylation in WT (Figure 1a). Strikingly, all remaining 19 established gASMs had at least one NORED region within the highest 29 ranked regions (FDR<6.8×10^−4^; Figure 2b and c and Supplementary Figure S2B).

As exemplified by gASMs of *Peg3*, *Inpp5f*, *Snrpn*/*Snurf*, *Kcnq1ot1*/*Kcnq1*, and *H19* from chromosome 7, NORED identified well-established gASMs (Figure 2d and f–i) and potential gASMs [46]. at *Cdh15* (Chr 8qE1) and *Nnat/Blcap* (Chr 2qH1) (Table 1). At *Peg3*, NORED detected two regions overlapping the known gASM (gray bar in Figure 2d): one 646 bp region that aligns with the established gASM start site and a second 4.2 kb region that covers the majority of the 4.5 kb gASM, but extends beyond the end site (Figure 2d). At *Inpp5*, a single 1.1 kb NORED region covers the majority of the 1.4 kb gASM (Figure 2f). Our NORED analyses defined two tandem ASMs of the *Snurf/Snrpn* locus as 2.1 and 3.3 kb, respectively (Figure 2g). Three NORED regions of 1.7, 116 and 1.6 kb were detected at *Kcnq1ot1*/*Kcnq1*, extending beyond the 2.1 kb gASM (Figure 2h). *H19* had a 2.4 kb NORED region within the larger reported gASM (7.3 kb; Figure 2i). NORED regions were also detected for *Rasgrf1:* one small (104 bp) within the 8.0 kb gASM and one larger 2.0 kb region extending beyond the end site of the gASM (Table 1 and Supplementary Table S2).

Additional regions near, but not overlapping with known gASMs were discovered at *Mest* and *Gtl2* (*Meg3*; Figure 1b and c, Table 1). NORED also identified additional imprinted ASM that are not considered gASM [48], such as somatic ASM at *H19* promoter (774 bp; Figure 2i). Furthermore, we identified a 1.8 kb NORED region near *Gab1*, which is reported to have imprinted gene expression; [49]. however, no gASM close to this region on Chr 8qC2 has been reported (Table 1). Finally, NORED identified regions with unknown imprinting or ASM status, as exemplified by the 178 bp, 1.1 kb, 170 bp, and 91 bp intergenic regions between *Gipr* and *Eml2* (Figure 2e). This locus is within Chr 7qA3 and the nearest known gASM (Peg3/Usp29) is upstream within Chr 7qA1; the nearest downstream known gASM (Snrpn/Snurf) is within a Chr 7qB5. The boundaries of known ASMs and other NORED regions are presented in Figure 2, Table 1, and Supplementary Table S2.

### Develop a new algorithm for genome-wide detection of bimodal methylation

As described above, identified NORED regions include known imprinted gASMs, which have bimodal methylation patterns. To characterize genomic regions that have potential to exhibit allelic methylation patterns, we sought a genotype-independent approach that could be applied in homozygous WT ESCs such as J1 ESC line. The bimodal distribution of methylation patterns has long been used in conventional bisulfite Sanger sequencing to validate ASMs. Stimulated by the concept and experimental design, we implemented a computational program, MethylMosaic, to detect the bimodal methylation patterns that are characteristic of imprinted ASMs. Notably, approaches based on the same concept that have successfully identified ASMs [50, 51]. Because there was no software for public use from earlier reports, we developed our own algorithms and used semisimulated data to assess the sensitivity and specificity of our approach (see details in Materials and Methods).

To identify bimodal regions by ‘MethylMosaic,’ we first calculated the read-level methylation and then the proportion of hypomethylated reads (hypomethylation index) and the proportion of hypermethylated reads (hypermethylation index) around each CpG site within the mouse genome. We then determined the true positive rate (TPR; 21 well-characterized gASMs considered as true positives) for various cutoffs of hypo- and hypermethylation indices to identify bimodal CpG sites (see details in Materials and Methods). To calculate the FPR, we simulated 10 null data sets by shuffling the methylation calls among reads at CpG positions. Importantly, the randomization of methylation among reads at each CpG site has the potential to alter read-level methylation, but keeps the CpG-level methylation intact. We calculated hypo- and hypermethylation indices and applied cutoffs (as described above) to identify bimodal CpG sites for null datasets, which were considered false positives. The FPR for each cutoff range was determined by averaging the FPR from null data sets. On the basis of the ROC curve, we selected the range from 0.2 to 0.75 as the bounds for hypermethylation and hypomethylation indices (Supplementary Figure S3A). For the WT dataset and the null data sets, we clustered individual CpGs into regions and ranked regions by the number of CpG sites. Null data sets were used to determine region-level FDR.

**Figure 3:**
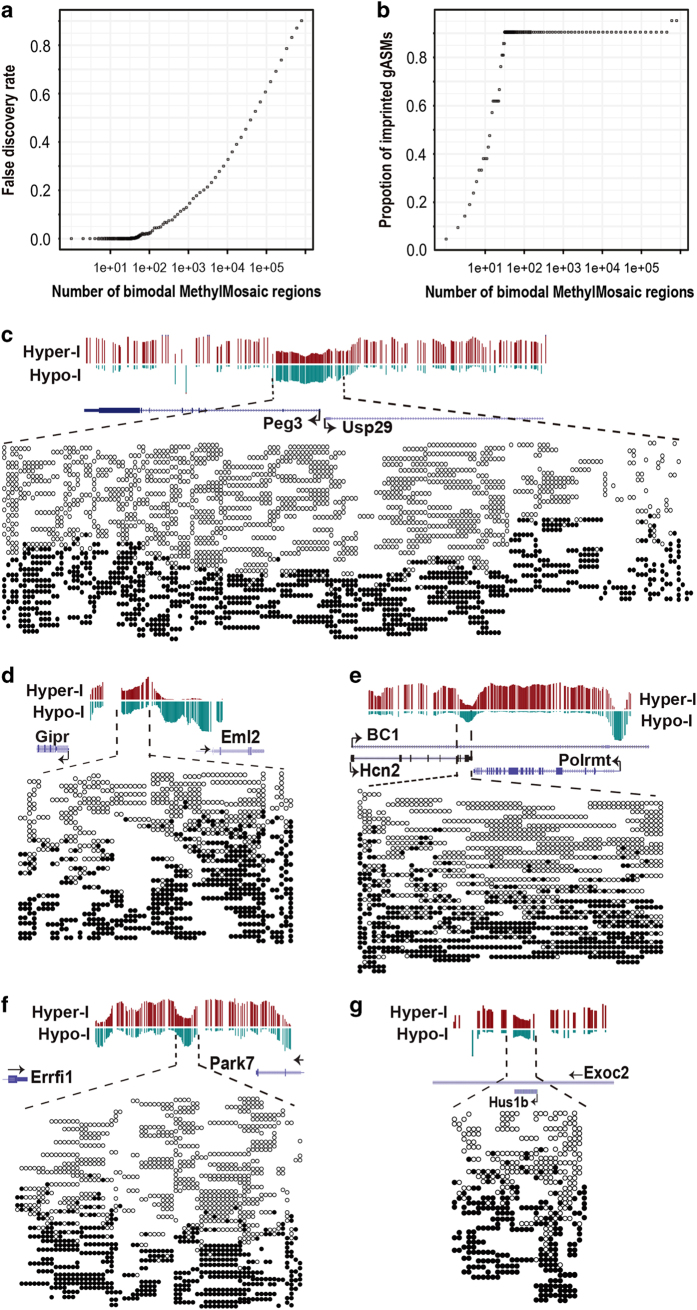
Read-level methylation reveals the bimodal distribution of hyper- and hypomethylated reads at gASMs and other NORED loci. (**a**) Number of bimodal methylation regions detected by MethylMosaic at different False discovery rate (FDR) values. FDR (*y* axis) as a function of rank (*x* axis) for bimodal regions. (**b**) Proportion of well-established gASMs (*y* axis) identified by MethylMosaic as a function of rank (*x* axis) for NORED regions. Nineteen gASMs rank among top 32 MethylMosaic regions. (**c**) MethylMosaic of known *Peg3* gASM demonstrates the robustness of the approach. *Peg3* has 674 reads, of which 427 have at least three CpGs. (**d**–**g**) MethylMosaic analyses reveals bimodal distribution of hyper- and hypomethylated reads at NORED regions that are not established gASMs. (**d**) *Gipr*/*Eml2* has 248 reads, of which 151 have at least three CpGs. (**e**) *Hcn2/Polrmt* has 163 reads, of which 126 have at least three CpGs. (**f**) *Errfi1*/*Park7* and has 254 reads, of which 168 have at least three CpGs. (**g**) *Hus1b*/*Exoc2* has 146 reads, of which 83 have at least three CpGs. (**c**–**g**) Hypermethylation Index is abbreviated as Hyper-I and Hypomethylation Index is abbreviated as Hypo-I (see Materials and Methods). Filled in circles (**•**) represent methylated CpGs and open circles (**○**) represent unmethylated CpGs. CpGs are clustered by read such that consecutive CpGs (with no horizontal gap) come from a single read, whereas, a gap indicates a separate read.

MethylMosaic analyses identified 2 487 regions as bimodal at FDR=0.20 (Figure 3a, Supplementary Figure S3B, Supplementary Table S3). Consistent with NORED, neither *Slc38a4* nor *Gnas 1A* were identified as bimodal, presumably due to low methylation in WT (Figure 1a). All remaining 19 established gASMs were among the highest 32 ranked regions (FDR<3.85×10^−3^; Figures 3a and b and Supplementary Figure S3B). Of *Peg3/Usp29* ASM, 166 consecutive CpG sites met criteria for hyper- and hypomethylation index cutoffs. From overlapping reads of CpG-centered windows, we retrieved 674 reads covering 169 CpG sites; 427 of those reads contained at least three CpG sites per read (Figure 3c). Compared to 20–40 PCR clones in Bisulfite Sanger sequencing, hundreds of reads (up to 674 reads here) demonstrate the robustness of MethylMosaic. MethylMosaic also revealed bimodal distribution of hyper- and hypomethylated at *Gipr*/*Eml2*, *Hcn2*/*Polrmt*, *Errfi1*/*Park7* and *Hus1b*/*Exoc2* (Figure 3d–g). At these four loci, we retrieved 151, 126, 168 and 83 reads having at least three CpG sites from 248, 163, 254 and 146 total reads, respectively.

### Characterization of genomic loci that are NORED and have bimodal methylation

As expected, both NORED and MethylMosaic identified 19 well-characterized gASMs. To characterize the extent to which other NORED regions have potential to exhibit allelic methylation patterns, we compared ~2 500 regions presented for each method. Comparison demonstrated that only 8.4% (207) of 2468 NORED regions overlapped at least one bimodal region and only 2.2% (152) of 2487 bimodal regions overlapped at least one NORED region (Supplementary Figures S4A and B). Therefore, the majority of NORED regions were not bimodal (Supplementary Figure S4A).

To rule out the possibility that the low rate of NORED that are also bimodal is driven by the number of regions presented for each method, we determined the proportion of bimodal NORED regions at multiple FDRs for MethylMosaic. For 2468 NORED presented in Supplementary Table S2, bimodal NORED regions would not become the majority of total NORED regions until MethylMosaic FDR=0.74. For the 207 NORED (FDR=0.005) regions presented in Table 1, 50.2 to 71.5% would be considered bimodal between FDR=0.42 and FDR=0.90 for MethylMosaic. We therefore conclude that NORED and MethylMosaic are independent, but not mutually exclusive, methods for identifying genomic regions with specific DNA methylation characteristics. That is, gASM are both NORED and bimodal; however, there are indeed other genomic regions that are either NORED or bimodal, but not both.

Of the top 207 ranked NORED regions presented in Table 1, 75 (36.2%) were bimodal. In addition to gASMs, NORED regions that had corresponding bimodal regions included *Gipr*/*Eml2*, *Hcn2*/*Polrmt*, *Errfi1*/*Park7* and *Hus1b*/*Exoc2* (Figures 3d–g). Note, *Hus1b* ASM is within the intron of *Exoc2* gene. NORED regions at possible gASM of *Nnat/Blcap* and imprinted *Gab1* were also identified as bimodal, whereas potential gASM at *Cdh15* was not (Table 1).

### Characterization of genes associated with regions that are both NORED and bimodal, exclusively NORED or exclusively bimodal

Using Chemical and Genetic Perturbations (CGP) gene sets from MSigDB (see Materials and Methods), we first asked whether genes associated with 2 468 NORED regions and genes associated with 2 487 bimodal regions were enriched in imprinted genes. NORED regions were enriched for imprinted genes (27 genes, *q*=2.03×10^−21^), whereas, bimodal genes were not reported within the top 100 enriched gene sets (Supplementary Tables S4 and S5). MethylMosaic regions were enriched for the gene set of high CpG density promoters bearing both H3K4me3 and H3K27me3 histone modifications (167 genes, *q*=1.80×10^−53^; Supplementary Table S5). Notably, for genes associated with 207 regions considered both NORED and bimodal, imprinted genes was the top gene set identified (19 genes, *q*=5.84×10^−31^; Supplementary Table S6).

To provide insight into potential functional implications for regions detected separately or by both NORED and MethylMosaic, we identified enrichment of CGP gene sets for 123, 1 427 and 1 627 gene identifiers associated with 207 bimodal NORED regions, 2 261 exclusively NORED regions, and 2 335 exclusively MethylMosaic regions, respectively. Other than imprinted genes, disease-relevant gene sets identified for bimodal NORED-associated genes included nasopharyngeal carcinoma (22 genes, *q*=5.28×10^−06^), genes upregulated in mutated KRAS lung cancer model (10 genes, *q*=7.69×10^−4^), pancreatic cancer (6 genes, *q*=4.05×10^−3^), TP53 targets (11 genes, *q*=2.24×10^−2^), Alzheimer's disease upregulated genes (13 genes, *q*=3.07×10^−2^), female fertility (3 genes, *q*=9.22×10^−3^) and metabolic syndrome (11 genes, *q*=2.53×10^−2^; Supplementary Table S6).

NORED exclusive-associated genes were also enriched nasopharyngeal carcinoma (163 genes, *q*=1.92×10^−31^), TP53 targets (113 genes, *q*=6.18×10^−24^), Alzheimer’s disease upregulated genes (146 genes; 1.98×10^−26^), and metabolic syndrome (99 genes, *q*=4.06×10^−16^; Supplementary Table S7). Enrichment for genes hypermethylated in liver cancer (96 genes, *q*=4.96×10^−22^), lung cancer (43 genes, *q*=5.41×10^−11^), and lymphoma tumors of transgenic mice (16 genes, *q*=9.23×10^−10^) was observed only in NORED exclusive-associated genes (Supplementary Table S7). Furthermore, NORED exclusive-associated genes were uniquely enriched in genes characterized by H3K27me3 with polycomb proteins (SUZ12 or EED) bound to promoters that experience *de novo* DNA methylation in cancers (18 genes, *q*=6.94×10^−9^; Supplementary Table S7).

Similar to bimodal NORED and NORED exclusive regions, MethylMosaic exclusive-associated genes were also enriched nasopharyngeal carcinoma (149 genes, *q*=1.07×10^−19^), TP53 targets (126 genes, *q*=3.13×10^−26^), Alzheimer's disease upregulated genes (205 genes, *q*=9.61×10^−52^), and metabolic syndrome (125 genes, *q*=1.31×10^−24^; Supplementary Table S8). Enrichment for genes upregulated in chronic myleogenous leukemia (151 genes, *q*=2.07×10^−32^) and upregulated in uveal melanoma (104 genes, *q*=8.85×10^−29^) were uniquely identified for MethylMosaic exclusive regions (Supplementary Table S8).

### Independent characterization of genomic regions with bimodal methylation patterns with newly generated ESC lines

We next aim to further examine regions with bimodal methylation patterns with experiments. Four scenarios could explain a genomic region bearing bimodal methylation patterns: (1) bona fide imprinted ASM (that is, parent-of-origin dependent and genetics-independent); (2) genetics-dependent ASM but independent of parent-of-origin; (3) one allele with hypomethylation (or hypermethylation) in half of cells and the same allele with hypermethylation (or hypomethylation) in the remaining half of cells (named as switchable ASM); and (4) half of cells with biallelic hypermethylation and half of cells with biallelic hypomethylation (see illustration below).

To experimentally examine these identified MethylMosaic regions in the mouse genome, we sought hybrid ESC lines with single-nucleotide polymorphisms (SNPs) between two alleles for characterizations. We are interested in the characterization of genes with potential in neurological disorders. Therefore, we focused our validation on two ASMs: *Hcn2* with known roles in epilepsy, inflammatory and chronic pain [52], and *Park7* (or *DJ-1*) for Parkinson’s disease [53]. We first used several available ESC lines; however, we did not detect bimodal methylation patterns (data not shown). We reason that because these lines were generated many years ago, multiple passages might result in aberrant methylation patterns similar to loss of imprinting described in human ESCs [54]. In support of our reasoning, *H19*/*Igf2* ASM was frequently lost in ESC lines (confirmed, data not shown). Alternatively, ASMs might be transient [49], and the developmental stage of the inner cell mass might not be appropriate for ASMs. We therefore decided to generate our own mouse ESC lines from F1 hybrid mice (reciprocal cross between 129S1/SvimJ and Cast/EiJ or between C57BL/6J and Cast/EiJ).

**Figure 4:**
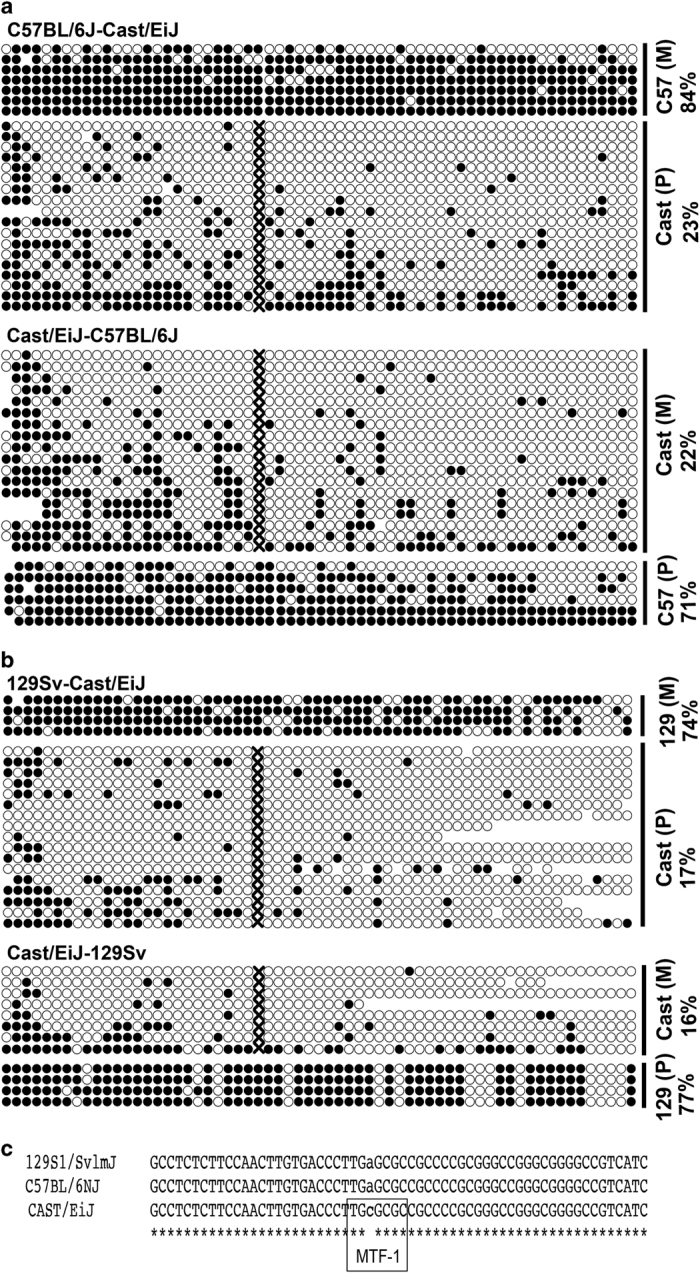
Bisulfite Sanger sequencing with four F1 hybrid ESCs confirmed the ASM at Hcn2/Polrmt locus and revealed the preferred hypomethylation of Cast allele. The ‘X’ indicates that a CpG site is not present in the allele(s). (**a**) The maternal (M) C57 allele and the paternal (P) Cast allele of a hybrid ESC line from a cross between C57 and Cast were hypermethylated and hypomethylated, respectively (top); The maternal Cast allele and the paternal C57 allele of a hybrid ESC line from a cross between Cast and C57 were hypomethylated and hypermethylated, respectively (bottom). (**b**) The maternal 129 allele and the paternal Cast allele of a hybrid ESC line from a cross between 129 and Cast were hypermethylated and hypomethylated, respectively (top); The maternal Cast allele and the paternal 129 allele of a hybrid ESC line from a cross between Cast and 129 were hypomethylated and hypermethylated, respectively (bottom). (**c**) DNA sequence alignment revealed a SNP-introduced binding motif for transcription factor MTF-1.

With DNA from two ESC lines (C57Cast and CastC57), we did bisulfite Sanger sequencing to examine methylation status of *Hcn2/Polrmt* ASM. We tried and succeeded with one pair of primers that cover 62 CpG sites, thereby enabling us to have better insight at the *Hcn2/Polrmt* ASM (Figure 4a). Indeed, our data revealed allele-specific DNA methylation patterns: the Cast allele (paternal) from the C57Cast line was hypomethylated (23% methylation), whereas the C57 allele (maternal) was hypermethylated (84% methylation; Figure 4a, top panel). Consistently, the Cast allele (maternal) from the CastC57 line was hypomethylated (22% methylation), whereas the C57 allele (paternal) was hypermethylated (71% methylation) (Figure 4a, bottom panel). While bimodal methylation patterns are expected according to our MethylMosaic analyses (Figure 3e), it is unexpected that the allele-specific methylation pattern would be independent of parent-of-origin. However, at the *Hcn2/Polrmt* ASM, the Cast allele was hypomethylated when it was maternally or paternally inherited.

To reinforce our confidence with our results above, we generated two more ESC lines from strains 129 and Cast (reciprocal hybrid cross), as biological replicates. Indeed, bisulfite Sanger sequencing data confirmed allelic methylation patterns. Within the 129Cast ESC line, the 129 allele (maternal) was hypermethylated (74%), and the Cast allele (paternal) was hypomethylated (17%); Within the Cast129 ESC line, the Cast allele was hypomethylated (16%) and the 129 allele was hypermethylated (77%; Figure 4b). Again, allele specificity of *Hcn2* ASM was independent of parent-of-origin. Therefore, we conclude that *Hcn2* ASM has bimodal methylation patterns, as revealed by MethylMosaic data in inbred J1 ESC line and bisulfite Sanger sequencing data in four independent ESC lines (129Cast, Cast129, C57Cast and CastC57), and that the Cast allele always has hypomethylated CpG sites, independent of parent-of-origin. Because of the latter, *Hcn2* ASM is not a bona fide imprinted ASM.

### One SNP variant of the Cast allele results in a binding motif for transcription factor at *Hcn2*/*Polrmt* ASM

The hypomethylated Cast allele promoted us to ask whether this biased hypomethylation was related to genetic variation. There are three SNP variants around *Hcn2* ASM. One variant (rs240718423: cytosine in Cast allele), intriguingly, results in an additional CpG dinucleotide. The resulting sequence of TG***C***GCGC becomes the core consensus sequence TGCRCNC (R=A or G, N=any nucleotide) of a metal regulatory transcription factor MTF-1 (Figure 4c). MTF-1 is a pluripotent regulator that regulates cell adaptation to various stress conditions (primarily exposure to heavy metal, and stresses of hypoxia and oxidative stress) [55, 56]. In contrast, TG***A***GCGC in the 129 or C57 alleles is not a binding motif for MTF-1. Whether this MTF-1 predisposes the Cast allele to low methylation during cycles of demethylation and methylation for the generation of *Hcn2* ASM, however, remains to be determined. Another variant (rs259784301: adenine in Cast allele), results in one less CpG dinucleotide in the Cast allele than in the 129 or C57 alleles (Figure 4a and b).

**Figure 5:**
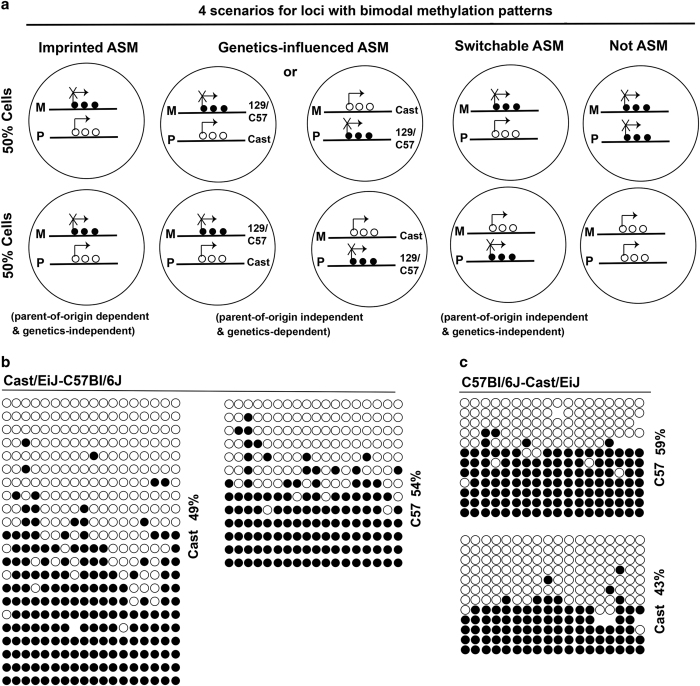
Four scenarios for regions with bimodal methylation patterns and Park7 ASM. (**a**) Four scenarios to explain genomic regions bearing bimodal methylation patterns: bona fide imprinted ASM; genetics-dependent ASM that is independent of parent-of-origin; one allele with hypomethylation (or hypermethylation) in half of cells and the same allele with hypermethylation (or hypomethylation) in the remaining half of cells; and half of cells with biallelic hypermethylation and half of cells with biallelic hypomethylation. (**b**) In hybrid CastC57 ESC line, the maternal Cast allele (left) had half highly methylated and half lowly methylated PCR clones; similarly, the paternal C57 allele (right) had half highly methylated and half lowly methylated PCR clones. (**c**) In hybrid C57Cast ESC line, the maternal C57 allele (top) had half highly methylated and half lowly methylated PCR clones; similarly, the paternal Cast allele (bottom) had half highly methylated and half lowly methylated PCR clones.

Multiple SNP variants may predispose the Cast allele to be hypomethylated, prompting us to examine a similar possibility at the corresponding region in J1 (inbred 129 strain) ESCs. We did Sanger sequencing of PCR amplified products. As expected, the corresponding region did not contain any variants at known SNP positions or any *de novo* mutations (Supplementary Figure S5), ruling out the possibility of genetic variants in J1 ESCs at this locus. Therefore, we conclude that genetic variation is not necessary for bimodal methylation patterns at this *Hcn2* region, but the SNP-associated motif introduced in the Cast allele may predispose it to low methylation (Figure 4). On the basis of the mono-allelic methylation at *Hcn2* locus (revealed in four hybrid ESC lines), it is reasonable for us to speculate that bimodal methylation patterns at this locus in mouse inbred J1 ESC line (Figure 3e) and in human H1 ESC line (presented below) are ‘switchable’ ASM, as opposed to the scenario where half of the cells are biallelically methylated and the other half are biallelically unmethylated (see scenarios in Figure 5 below).

### Independent validations of Park7 ASM suggests a scenario of random, switchable ASM

Simultaneously, we have examined the *Park7* ASM (see bimodal methylation in Figure 3f) in four new ESC lines. The primers we used cover 18 CpG sites, and methylation status at the paternal allele or the maternal allele showed interesting patterns: Out of 35 PCR clones examined for the CastC57 ESC line, we found that 22 clones with half of them hypomethylated (or hypermethylated) were for maternal Cast allele and that 13 clones with half of them hypomethylated (or hypermethylated) were for paternal C57 allele (Figure 5b). In other words, each allele seemed to have equal chance to be hypermethylated or hypomethylated. Out of 24 PCR clones from an independent C57Cast ESC line, we found similar results: 12 clones with half of them either hypermethylated or hypomethylated were for maternal C57 allele; the remaining 12 clones with half of them either hypermethylated or hypomethylated were paternal Cast allele (Figure 5c). Bisulfite Sanger sequencing data from two biological replicates (two ESC lines; Cast129 and 129Cast) reproduced observations above (data not shown). These data suggested a random, switchable allele-specific methylation pattern. However, additional data such as single cell DNA methylomes are needed for conclusion.

Compared to *Hcn2* ASM, the genetic differences between the Cast allele and the C57 or 129 alleles at *Park7* ASM did not result in a new binding motif for a potential transcription factor (data not shown), thereby not predisposing one allele toward hypomethylation. Without a new binding motif, the maternally and paternally inherited alleles at *Park7* presumably behave similarly to *Hcn2* in inbred mouse J1 ESC line. The latter has no genetic variations. Collectively, we conclude that *Park7* ASM indeed shows bimodal methylation pattern, and that the differences between Cast allele and C57 (or 129) allele did not result in a preference of one allele for hypomethylation.

### Conserved ASM at *Hcn2/Polrmt* locus in the human genome

**Figure 6:**
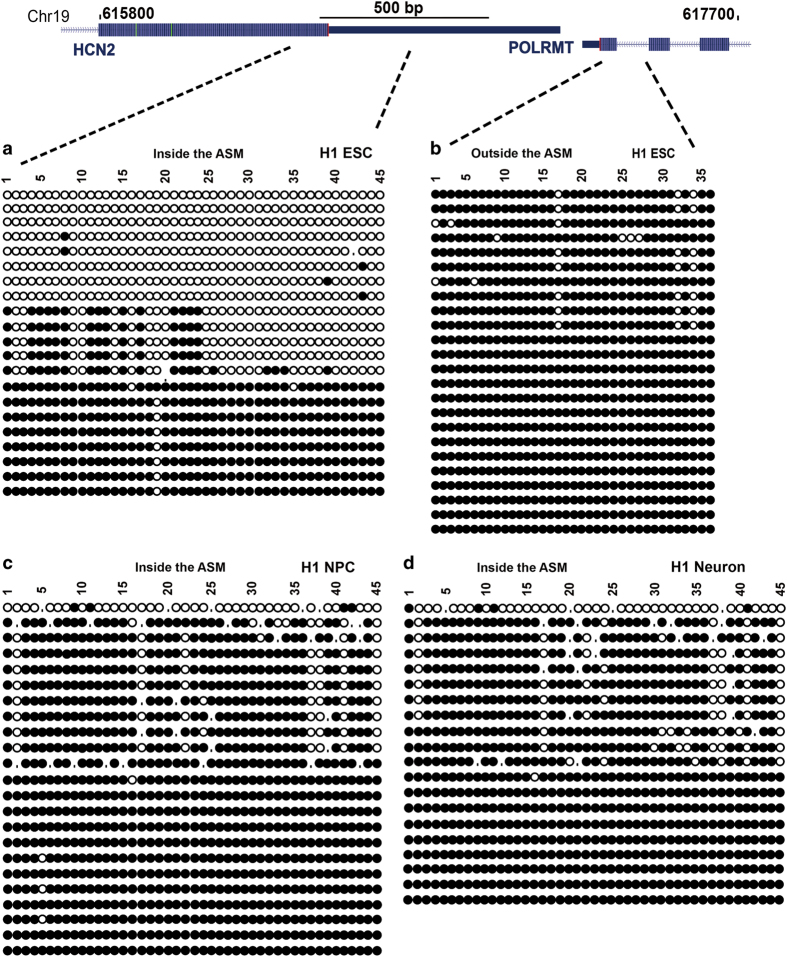
Bisulfite Sanger sequencing confirmed that transient DMR at *Hcn2/Polrmt* locus is conserved in the human genome. (**a**) Bisulfite Sanger sequencing revealed bimodal distribution of hyper- and hypomethylated reads. (**b**) Bisulfite Sanger sequencing revealed fully methylated reads from a neighboring control region. (**c** and **d**) Bisulfite Sanger sequencing revealed a *de novo* methylation process to resulting in hypermethylation of CpG sites from the hypomethylated allele upon differentiation of H1 ESCs into NPCs (**c**) or neurons (**d**).

Having demonstrated bimodal methylation patterns of *Hcn2/Polrmt* ASM in five independent mouse ESC lines (J1, 129Cast, Cast129, CastC57 and C57Cast), we next explored the evolutionarily conserved ASM at *Hcn2*/*Polrmt* locus in the human genome for further validation. Because bimodal patterns were detected in five mouse ESC lines, but a recent examination of cortical neurons from a hybrid cross (129×Cast) did not find ASM [57], we decided to examine human ESC line. In the human genome, the *Hcn2* and *Polrmt* genes have convergent genomic organization, similar to the mouse orthologues (Figure 6). We designed two pairs of primers with one pair inside of the predicted ASM and the other pair outside of the ASM (Figure 6). Strikingly, monoclonal sequencing reads with 45 CpG sites from the ‘inside’ pair have roughly half hypo- and half hypermethylated reads (Figure 6a). In contrast, all PCR clones from the ‘outside’ pair contain hypermethylated reads (Figure 6b). We conclude that *Hcn2* ASM is conserved in the human genome.

### The transient ASM at *Hcn2*/*Polrmt* locus participates in early embryonic development

The observation that hybrid cortical neurons did not have a bimodal methylation pattern at *Hcn2*/*Polrmt* locus [57]. (data not shown), suggests a role of *Hcn2* ASM during development. We then examined *Hcn2* ASM using *in vitro* differentiated neuron progenitor cells (NPCs) and neurons derived from H1 ESCs. Consistent with mouse cortical neurons, human NPCs and neurons became fully methylated, confirming the loss of the bimodal patterns at *Hcn2*/*Polrmt* locus upon differentiation (Figure 6c and d). Therefore, this ASM is transiently presented during early embryonic development.

## Discussion

### DNMT1-dependent methylation regions for mechanistic insights of disease susceptibility

As the predominantly expressed DNMT especially in somatic cells, DNMT1 is the favorite enzyme out of three DNMTs for investigation (for example, the development of DNMT1 inhibitor in the treatment of cancers), and DNMT1-dependent methylation patterns are presumably important for understanding disease pathogenesis. Herein we have used/developed two approaches to characterize DNMT1-dependent methylation patterns at genomic regions in the mouse genome. The NORED approach, which is a newly developed method in this study, identified 2 468 genomic regions dependent on DNMT1 function. Among them, 207 regions also show bimodal methylation patterns (that is, also MethylMosaic regions). Regions showing bimodal methylation patterns include 19 known imprinted gASMs. Relevant to human health, these gASMs and tissue-specific ASMs are particularly vulnerable to environment-induced perturbation [2, 26, 28]. Given that gASMs and novel MethylMosaic regions share the feature of allelic methylation patterns, it is reasonable to expect that these MethylMosaic regions would also be vulnerable to environmental exposure. Indeed, unpublished base resolution methylomes of endocrine disruptor-exposed mice reveal many such regions were impacted. We therefore expect that these NORED/MethylMosaic regions will be used extensively to inform studies in exposed mice in the future.

Many genes from both NORED and MethylMosaic regions have potential in human diseases. *Hcn2* (Figures 3e, 4 and 6) is responsible for hyperpolarization-activated cation (HCN) channel, which is linked to the generation of cardiac pacemaker depolarization and the control of neuronal excitability and plasticity. *Hcn2* is implicated in the pathogenesis of epilepsy [58, 59]. and linked to chronic pain as well [52]. *Polrmt* is involved in mitochondrial transcription [60]. *Park7(DJ-1)*/*Errfi1* locus is involved in Parkinson’s disease (Figures 3f and 5) [53, 61]. In addition to neurological activities, novel bimodal NORED regions that have potential roles in type 2 diabetes at the *Gipr*/*Eml2* locus (Figures 2e and 3d) [62]. and in cell cycle checkpoint at *Hus1b*/*Exoc2* locus (Figure 3g) [63]. were identified. Altogether, our data open new windows to markedly improve the understanding of many complex human diseases [64–67].

While bimodal regions were not reported as being enriched for imprinted genes (not within the top 100 enriched gene sets from CGP), they were enriched for genes identified as having both active H3K4me3 and inactive H3K27me3 histone modifications (within the top 5 enriched gene sets; Supplementary Table S5). This would be consistent with a scenario of allelic methylation, with an unmethylated allele having active histone marks and a methylated allele having inactive histone marks. Alternatively, it could be explained by a mixed cell population, with half of cells having active epigenetic marks and the other half having inactive marks. Notably, DNMT1-dependent regions were enriched for imprinted genes (within the top 20 enriched gene sets; Supplementary Table S4). Overall our results indicate that MethylMosaic identified bimodal regions, but imprinted genes are more apparent in NORED.

Further reinforcing the importance of recognizing DNMT1-dependent regions for disease, NORED exclusive regions uniquely identified genes that are known to become *de novo* DNA methylated in cancer and genes that have previously been reported as hypermethylated in cancer (Supplementary Table S7). Perhaps reflecting the complexity of neurodegenerative and metabolic disorders, NORED-exclusive, MethylMosaic-exclusive, and bimodal NORED were all enriched for Alzheimer’s upregulated genes and metabolic syndrome network genes (Supplementary Tables S6). Importantly, bimodal NORED regions were highly enriched in imprinted genes (highest ranked gene set) and uniquely enriched in female fertility genes, both of which could have implications ‘developmental origin of adult disease’ and ‘transgenerational epigenetic inheritance’ (Supplementary Table S6). Enrichment analysis of CGP reaffirmed our assertion that allelic methylation and DNMT1-depenence are separate characteristics that coincide at gASMs and additional bimodal NORED regions.

### NORED demarcates DNMT1-dependent methylation at ASMs

Knowing the exact genomic region of an ASM is important to diagnose and understand the pathogenesis of imprinting disorders, such as Prader-Willi and Angelman syndromes [68, 69]. Prior examination of patient samples with microdeletions to define the location of ASMs is time consuming and limited by the availability of patient samples [70]. Systematic identification of all ASM sizes and characterization of alterable CpG methylation sites within ASMs in the mouse genome will be important [57, 71–74]. The boundaries for DNMT1-dependent methylation of known ASMs and other NORED regions were presented in Figure 2, Table 1 and Supplementary Table S2. While the overall methylation at established gASM regions for both *Rasgrf1* and *H19* showed partial recovery in r1KO ESCs, both had smaller NORED regions, within the larger established gASMs, that were completely lost (and not rescued) in DNMT1-deficient cells (Figure 2a and i). Overall, this suggests two possible scenarios: (1) that only a subset of CpGs within germline imprinted loci are NORED or (2) that NORED accurately defines CpGs responsible for regulating parent-of-origin allelic methylation at imprinted loci. We favor the latter because it is consistent with the mechanistic understanding that DNMT1 is absolutely critical for maintaining parent-of-origin allelic DNA methylation at germline imprints [41, 47].

A mosaic is comprised of smaller subunits from which a larger pattern emerges. Analogously, our ‘MethylMosaic’ approach reveals DNA methylation patterns at a genomic region that is composed from whole genome bisulfite sequencing reads, based on the principles that each sequencing read represents a separate DNA molecule (see Materials and Methods). By evaluating read-level methylation, as opposed to CpG-level methylation, we observe emergent patterns at the molecular level that can be used to provide mechanistic insight. While many patterns could be explored, highly methylated and lowly methylated reads—which are quantified as hyper- and hypomethylation indices—have been presented here to detect genomic regions with bimodal methylation that occurs at ASMs (Figure 3 and Supplementary Table S3).

By combining DNMT1-dependent DNA methylation loss with bimodal methylation patterns characteristic of allelic methylation, we have presented regions that share epigenetic properties with gASMs. Unexpectedly, we identified additional regions (for example, *Park7* and *Hcn2*) that although both vulnerable and bimodal, do not display parent-of-origin dependent allelic methylation in reciprocal cross ESCs. While further detailed characterization of novel bimodal NORED regions is needed to examine parental and genetic influences on allelic methylation at other loci, we have validated parent-of-origin independent bimodal methylation of two loci, *Park7* and *Hcn2*.

### Cross talk between genetics and epigenetics via non-imprinted ASMs in the mammalian genome

Genetic variation has long been associated with common diseases. Over the past decades, numerous genome-wide association studies (GWAS) have been performed for many common diseases, including diabetes, autoimmune diseases, and neurological disorders [75]. Allele frequencies of hundreds of common variants are reported as statistically correlated with diseases. However, it is controversial whether these variants have biological relevance to disease pathogenesis and clinical prognosis or treatment [76, 77].

Contrary to early expectations of SNP variants disrupting protein coding genes, the vast majority (about 88%) of GWAS-identified SNPs reside in intergenic or intronic regions, which makes it difficult to interpret their functional relevance. Herein, our investigations provide a clue that a single nucleotide difference (rs240718423 in Cast allele; Figure 4c) could have functional relevance. This SNP variant may predispose Cast allele to be hypomethylated and the other allele to be hypermethylated at *Hcn2*/*Polrmt* ASM. While further investigation is needed to demonstrate the influence of *Hcn2*/*Polrmt* ASM on the expression of *Hcn2* and *Polrmt*, published data confirm that the region surrounding this ASM is associated with inactive marks including H3K9me3, H4K20me3 and H3K27me3 and the *Hcn2* promoter is associated with inactive H3K27me3 (see Figure 6 in [78].). These inactive marks are widely considered as inactive histone marks that repress gene transcription [3, 79, 80]. Because *Hcn2* is highly expressed in ESCs, these inactive marks are expected to be associated with the silent allele (presumably hypermethylated 129 or C57 allele, not the hypomethylated Cast allele). Note, the coverage of the published ChIP-seq data was not enough to call allelic chromatin marks [78]. Combined with previously reported findings, our results reveal a possibility that the difference at one single nucleotide could be amplified through alteration of local chromatin structure, thereby changing the fate of a gene on a given allele.

### A new hypothesis of regional ACI: an X-chromosome inactivation (XCI)-like mechanism in controlling autosomal genes in mammals?

Not all genes on the inactivated X chromosome are silenced. In other words, it is a mechanism of regional inactivation of X chromosome. The selection of inactivated X chromosome in eutherian (placental) mammals, such as mice and humans, involves the transcription of a master regulator Xist (X-inactive specific transcript), a long non-coding RNA, as well as the expression of antisense Tsix of Xist. DNA methylation and inactive histone modification marks are necessary for the inactivation of selected X chromosome.

The feature above resembles that of *Hcn2*/*Polrmt* ASM and other ASMs. Methylation of CpG sites happens at one allele (for example, C57 or 129), but not the other Cast allele at the *Hcn2*/*Polrmt* locus. Inactive histone modification marks including H3K9me3, H4K20me3 and H3K27me3 occupy the ASM and regions beyond the ASM [78]. Given the fact that *Hcn2* is highly expressed in ESCs (in report [78]. and our own data) and that these three inactive marks are associated with silent genes [79, 81], it is reasonable to expect that C57 or 129 allele (when combined with Cast allele) was the repressed allele. In addition, our unpublished observation suggests that *Hcn2*/*Polrmt* ASM- (and other ASMs) associated repressive chromatin structure extend beyond ASM region. In other words, the ASM-associated repressive chromatin controls the expression of genes over a long genomic region, resembling the inhibition of genes by repressive chromatin on inactivated X chromosome. The expected switchable feature of some ASMs (suspected for *Hcn2*/*Polrmt* ASM in mouse J1 and human H1 ESC lines; *Park7* ASM in four tested mouse ESC lines) resembles the observation of random inactivation of one of the two X chromosomes. Lastly, *Hcn2*/*Polrmt* locus also shares the feature of having a long non-coding RNA, BC1. Whether BC1 plays a similar role as Xist/Tix in the case of inactivation of one X chromosome or as Kcnq1ot1 in the case of repression of non-imprinted allele at *Kcnq1*/*Kcnq1ot1* locus, however, requires further investigation. All together, these shared features prompt us to propose a hypothesis that a regional autosomal chromosome inactivation (ACI), like XCI mechanism, may control some autosomal genes. We envision that this ACI acts regionally (that is, controlling only a subset of genes on a given autosome; not entire chromosome) and that these autosomal genes would be centered around the identified bimodal MethylMosaic ASMs. A close concept of ‘parallel to XCI’ is also recently proposed [82]. The mechanism of ACI and non-imprinted monoallelic methylation may provide alternative explanations for diseases such as DiGeorge syndrome, a common hemizygous microdeletion syndrome [83].

## Materials and Methods

### Cell culture, DNMT1-rescued 1KO cells, and human ESC differentiation

Mouse ES cells were cultured as described before [42]. Briefly, mouse ES cells (J1, 1KO, DKO, TKO) were maintained without feeder cells on 0.1% gelatin coated Petri dish in DMEM medium supplemented with 15% FBS (ES cell grade), 2 mM glutamine, 10 uM mercapto-ethanol, 100 U ml^−1^ LIF, Penicillin/Streptomycin mixture 100 μg ml^−1^, 1× non-essential amino acid. Cultured DNMT1 KO (1KO) cells were transfected with constructs expressing GFP-fused DNMT1 (kind gift of Dr Heinrich Leonhardt [84].). DNMT1-GFP-rescued 1KO cells were sorted using facility of Bloomberg School of Public Health. Note that J1 ES line was generated from inbred 129 strain.

Four hybrid ES cell lines were directly derived from the blastocysts, which were from the reciprocal crosses between mice on different genetic backgrounds (for example, Cross between Cast/EiJ and 129S1/SvimJ or cross between Cast/EiJ and C57BL/6J). Primary mouse embryo fibroblasts inactivated by Mitomycin C were used as feeder cells. ES Cells were expanded on pre-coated plates with 0.1% Gelatin in LIF (+) medium on feeder cells and then moved to feeder-free 2i medium (EMD Millipore, Temecula, CA, USA) to get rid of feeder cells.

The differentiation of human H1 ESCs (Wi Cell, Madison, WI) into neuronal progenitor cells (NPCs) and neurons were performed similarly using our developed RONA (rosette-type neural aggregates) method [85, 86]. Briefly, detached hESC colonies were grown in suspension in human ES cell medium without FGF2 (defined as knockout serum replacement medium) in low attachment six-well plates (Corning, Corning, NY, USA), supplemented with Noggin (50 ng ml^−1^; R&D systems, Minneapolis, MN, USA) or Dorsomorphin (1 μM, Tocris Bioscience, Bristol, UK) and SB431542 (10 μM, Tocris Bioscience) from day 2 to day 6. Free-floating embyroid bodies (EBs) were attached and supplied with N2-induction medium (NIM) containing DMEM/F12 (Invitrogen, Carlsbad, CA, USA), 1% N_2_ supplement (Invitrogen), 100 μm NEAA (Invitrogen), 1 mM Glutamax (Invitrogen), and heparin (2 μg ml^−1^; Sigma, St Louis, MO, USA) from day 7 to day 16. Highly compact 3D column-like neural aggregates were collected and maintained as neurospheres in Neurobasal medium containing B27 minus vitamin A (Invitrogen), 1 mM Glutamax 1 day. For neuronal differentiation, dissociated neurospheres were maintained in neural differentiation medium containing Neurobasal/B27 (NB/B27, Invitrogen), BDNF (20 ng ml^−1^, PeproTech, Rocky Hill, NJ, USA), GDNF (20 ng ml^−1^, Peprotech), ascorbic acid (0.2 mM, Sigma), dibutyryl cAMP (0.5 mM, Sigma).

### Bisulfite whole-genome sequencing (BS-seq) library construction

One to five micrograms of genomic DNA of DNMT1-rescued 1KO cells was fragmentized to 200~500 bp by Covaris S2 sonicator (Covaris, Woburn, MA, USA). End repairing was then performed following manufacturer’s instruction (End-It DNA end repair kit, Epicentre, Madison, WI, USA). After Ampure XP (Sigma) purification, adenine was added to 3′ end with 3 ml DNA Taq polymerase (M0267S, NEB) and 1 mM dATP in 50 ml reaction solution incubated at 70 °C for 30 min. After Ampure XP purification, 1 ml of Illumina Trueseq adaptors were ligated with 4 ml T4 DNA ligase (M0202L, NEB) in 40 ml reaction solution and incubated at 16 °C overnight. Fragments at 300–600 bp from adaptor-ligated DNA were collected from 2% agarose gel, and then bisulfite-treated using Imprint DNA modification Kit (MOD50-1 KIT, Sigma) as manufacturer instructed. PCR enrichment was performed to amplify the libraries, which were then collected from 2% agarose gel electrophoresis at size 300–600 bp. Hi-seq 2000 was used for generating all deep-sequencing data.

### Data processing of BS-seq data

Trim Galore version 0.4.0 using Cutadapt version 1.8.1 and FastQC version 0.11.2 was used to trim sequencing reads (http://www.bioinformatics.babraham.ac.uk/projects/trim_galore/). Bismark version 0.14.5 [87]. implementing Bowtie2 version 2.2.5 [88]. was used to align trimmed reads to using options -N 1 -D 20 -R 3 -X 1000—chunkmbs 1024. To generate a strain-specific reference genome for J1 and J1-derived ESCs, we substituted mouse strain 129S1 SNPs from Mouse Genomes Project [89]. SNP Release version 5 (REL-1505-SNPs_Indels) into GRCm38/mm10 genome. Bismark deduplication was used to remove PCR duplicates from aligned pairs and Bismark methylation extractor was used to determine the methylation status of cytosines To prevent methylation bias at the ends of reads, we removed methylation calls in the first eight base pairs of each read [87]. R version 3.2.2 was used for post-processing analyses. We merged CpG methylation calls on positive and negative strands into single, destranded CpG sites.

### Methylation of 21 well-characterized germline imprinted ASMs (gASMs)

Well-characterized gASMs were defined as the 21 gASMs identified in common between two sources [45, 46]. (Supplementary Table S1). Methylation across a gASM was calculated as the average of all covered CpG sites. Comparison of methylation in WT, 1KO, DKO, and TKO at these loci was determined by pairwise Wilcoxon rank-sum tests (paired) and *P*-values were adjusted for multiple comparisons by Bonferroni method.

### No restored DMRs (NORED)

The concept for NORED is that in order for a CpG site to be considered not restorable in DNMT1-rescued 1KO cells it would need to meet three criteria: (1) that it has sufficient methylation in WT; (2) that it experiences near complete methylation loss in 1KO; and (3) that it recovers minimum methylation in r1KO. To determine methylation levels in WT, 1KO and r1KO ESCs to use as cutoffs for these criteria, we performed an ROC-like analysis using a permutation-based approach. Only CpG sites from autosomal chromosomes with at least 5× coverage in each cell type (WT, 1KO, and r1KO ESCs) were used. To estimate the false positives at various combinations of methylation cutoffs for WT, 1KO, and r1KO, we simulated twenty null datasets by swapping the cell type labels at each CpG site among the three ESCs. For WT, we tested 0.25 and 0.30 as minimum values. For 1KO, we tested 0.03 and 0.05 as maximum values. For combinations of WT and 1KO, we tested r1KO between WT and 1KO at increments of 0.005 for maximum values. We applied these criteria to null data sets and considered all positives identified as false positives and all negatives as true negatives. FPR for each null dataset was calculated as the number of false positives divided by the sum of false positives and true negatives. FPR presented is the average FPR for twenty null datasets. We then applied the same cutoff combinations (described above) to the original data to identify positives and negatives. At FPR of 0.01, 70% of CpG sites within gASMs (defined as those in common from two sources [45, 46].) had at least 25% methylation in WT, at most 5% methylation in 1KO, and at most 12.5% methylation in r1KO; genome-wide 3.3% of CpG sites met these criteria (Supplementary Figure S2A). We therefore chose these values as the criteria to use to identify non-restored methylation at CpG sites.

To identify NORED regions, we developed a simple scoring system to combine nearby non-restored CpG sites into larger regions, allowing for an occasional restored CpG site to be included. At each CpG site, we assigned two points if it met the criteria and one point for each of the two proceeding and two following CpGs that met the criteria, for a maximum point of six points per CpG position. We then clustered individual CpG sites in to regions with the regionFinder function from Bump Hunter [90], using a cutoff of three points for inclusion. Cumulative score was used to rank regions, thereby taking into account both the number of CpG sites and the proportion of CpG sites within the region that meet the criteria. To determine the FDR for NORED, we applied the scoring and clustering algorithms to the permutated datasets to identify false positive regions. For each unique cumulative score in the original data, we calculated the number of regions considered positive at this threshold in the real data and in each null dataset. We then divided the number of false positives by the number of positives to calculate the FDR for each permuted dataset. The average FDR of twenty null datasets is presented as the FDR for the method.

### MethylMosaic

In diploid organisms, gASM is characterized by one allele being methylated and the other allele being unmethylated. Conventional sequencing of PCR clones to confirm gASM prompted us to develop MethylMosaic. It is expected that roughly half of sequencing reads (or tags) from these regions carry high proportion of methylated CpG sites, while the other half carry high proportion of unmethylated sites, leading to a bimodal distribution of methylation at these regions. For regions without gASM, all sequencing reads should have similar proportions of methylated CpG sites since the two parental chromosomes would have equivalent methylation level at these loci.

To calculate read-level methylation for WT ESCs, the methylation of each CpG site within a sequencing read was determined by comparing read sequence with reference genome where a conversion from Cytosine on reference genome to Thymine on read sequence indicates unmethylated status and no such conversion indicates methylation. The overall methylation of a read is calculated by dividing the number of methylated CpG sites by the total CpG sites covered by the read.

Hypomethylation and hypermethylation indices were introduced to identify bimodal regions. For each CpG site on autosomal chromosomes in the mouse genome, a window enclosing 300 bp upstream and 300 bp downstream of that site was defined. Sequencing reads overlapping each window were retrieved and hypomethylation index was calculated as the proportion of reads with at most 10% methylation. Similarly, hypermethylation index was calculated as the proportion of reads with at least 90% methylation. To select values of hypo- and hypermethylation indices to use as criteria to consider a CpG site bimodal, we applied various cutoff ranges (combinations of upper and lower bounds) to both hypo- and hypermethylation indices. We calculated the TPR as the proportion of 21 well-characterized gASMs, defined as those in common from two sources [45, 46], identified for each cutoff range. To calculate the FPR, we simulated 10 null datasets by shuffling the methylation calls at each CpG site among the reads that cover it. Notably, the randomization of methylation calls among reads at each CpG site has the potential to alter read-level methylation only. That is, the methylation at a given CpG site remains the same as it was prior to randomization. We calculated hypo- and hypermethylation indices and applied cutoff ranges (as described above) to identify the positive CpG sites within the null datasets (as described above). Because all positive CpG sites in the randomized data sets were considered false positives, we calculated the FPR for each cutoff range as the number of identified CpG divided by the number of CpG sites in the genome. The FPR was determined for each cutoff range by averaging the FPR from 10 null data sets. We selected 0.2 for the lower bound and 0.75 for the upper bound as criteria for both hypermethylation and hypomethylation indices based on the ROC curve (Supplementary Figure S3A). That is, we considered a CpG site to be bimodal if the proportion of hypomethylated reads within the defined window was at least 0.2 and at most 0.75 and the proportion of hypermethylated reads within the defined window was at least 0.2 and at most 0.75.

To cluster individual CpG sites into regions, we combined consecutive bimodal CpGs. For WT data, we ranked bimodal regions by the how many CpG sites were included. For each unique rank (ties were assigned to lowest rank), we determined the number of regions that would exceed the threshold for quantity of CpG sites within the region (that is number of regions considered positive at each rank). For each of the randomized data sets we combined individual false positive CpG sites into regions and calculated false positive bimodal regions as the number of regions exceeding the CpG site quantity threshold for a given rank of WT data. To calculate the FDR for each rank, we divided the number of false positive bimodal regions by the number of regions identified as positive in the WT data. FDR was calculated for each null data set and averaged to determine the region-level FDR for bimodal regions by MethylMosaic method.

### Enrichment analyses for chemical and genetic perturbations

MSigDB version 5.2 was used to identify overrepresented gene sets for Chemical and Genetic Perturbations from Curated Gene Sets at an FDR<0.05 [91].. For each region set, all genes overlapping the center of the region, with a transcription start site within ±100 bp from the center of a region, or nearest the gene (if region is intergenic and does not overlap gene annotations) were considered to associated with a region.

### Bisulfite sanger sequencing

DNA was extracted from four hybrid ESC lines, H1 ESCs, H1-differentiated NPC/neurons using phenol/chloroform/isoamyl alcohol (25:24:1). About 10 μg of those DNA was bisulfite converted and purified by Sigma Aldrich’s Imprint DNA Modification Kit (St Louis, MO, USA) following manufacturer’s protocol. Exact 2 μl of the bisulfite converted DNA was used as template for PCR amplification with KAPA HiFi HotStart Uracil Plus (Boston, MA, USA) used for the PCR reaction. For *Hcn2* ASM amplification, the primer sequences were as follows: forward, 5′- GGTGTAGTAGGTAGA GTTTGGTTAG-3′ and reverse, 5′- CTCAAAAATCACAAATTAAAAAAAA were used to amplify a 529 bp fragment. For *Park7* ASM, the primer sequences were as follows: forward, 5′- TTTAGGTGAATTTTTGGAATTGTTT-3′ and reverse, 5′- CCTTCCCTAACTACTTAAATTAACAC-3′ were used to amplify a 334 bp fragment. Amplicons were ligated to PMD19 vectors (Clonetech) with T4 DNA ligase (M0202L, NEB) and transformed into DH-5α competent cells (NEB). Monoclonal bacteria colonies carrying the plasmid were cultured and picked up from plates with AMP and X-gal. All the colonies were sequenced at the Genewiz (Boston).

For H1, H1-derived NPCs, and H1-derived neurons, primers based on conserved sequence of mouse genome: forward, 5′- GAGATGGTGTAGTAGGT-3′ and reverse, 5′- ACCAAATATTACACTTAAAAAA-3′ were used to amplify a 977 bp fragment inside of the potential DMR in the human genome for HCN2 gene. Amplicons were ligated to TA vectors from PCR cloning Kit (Invitrogen) and transformed into DH-5α competent cells. Monoclonal bacteria colonies carrying the plasmid were cultured and picked up from plates with AMP and X-gal. All the colonies were sequenced at the Beckman Genomic Com (Danvers, MA, USA). Sequenced reads were aligned back to the Bisulfite converted genome sequences by Seqman pro (Lasergene). PCR replicates were discarded and methylation for monoclonal reads was methylation was determined with BiQ analyzer [92].

## Figures and Tables

**Table 1:** Identification, demarcation, and characterization of highest ranked NORED regions

Chromosome location	Nearest gene(s)
chr1:33162585-33162912	*Khdrbs2/Prim2*
**chr1:63199813**-**63200708**	Gpr1/Zdbf2*
chr1:93825020-93825457	*D2hgdh*
chr1:93825898-93826406	*D2hgdh*
chr1:131148330-131149072	Eif2d/Dyrk3*
chr1:134328756-134329228	*Ppfia4*
chr2:28370200-28370811	*Ppp1r26/Olfm1*
chr2:31304679-31305167	*Ncs1/Ass1/*Gm5424
chr2:32630974-32631585	*Ak1*
chr2:34671610-34671792	*Gapvd1/Mapkap1*
chr2:39806681-39806881	*Ppp6c/Lrp1b*
chr2:59348096-59348285	5330411J11Rik
chr2:105511256-105512346	*Rcn1/Pax6os1*
chr2:130365512-130365714	*Cpxm1*
chr2:131044421-131045271	*Gfra4/Adam33*
**chr2:152686347-152686904**	Mcts2/H13*
**chr2:152686927-152687288**	Mcts2/H13*
chr2:157559602-157560417	Nnat/Blcap*
chr2:157561077-157561984	Nnat/Blcap*
chr2:158614669-158614805	*Slc32a1*
chr2:162485692-162485820	*Ptprt*
chr2:166522472-166522714	*Prex1/*5031425F14Rik
**chr2:174293692-174298026**	Nespas/Gnas*
**chr2:174299016-174300450**	Nespas/Gnas*
**chr2:174298047-174298914**	Nespas/Gnas*
**chr2:174300469-174300620**	Nespas/Gnas*
chr2:181307114-181307303	*Stmn3*
chr3:3194456-3199479	*Hnf4g/Cr2*
chr3:94413377-94413565	*Tdrkh*
chr4:43629280-43629987	*Npr2/Rgp1*
chr4:43992511-43993691	*Ccin/Clta*
chr4:93044119-93048203	*Tusc1/Izumo3*
chr4:121052686-121053078	*Col9a2*
chr4:136225076-136225355	Asap3*
chr4:138677545-138677765	*Ubxn10/Vwa5b1*
chr4:139142929-139143492	*Minos1/*Gm16287
chr4:140814187-140814664	*Gm13032/Padi1*
chr4:140869176-140869307	4930515B02Rik*/Padi1*
chr4:145514859-145516993	Gm13212*
chr4:147808990-147809826	Gm13157*
chr4:150039937-150040661	*Mir34a/H6pd*
chr4:150879968-150880071	Errfi1/Park7*
chr5:3733077-3733199	*Ankib1*
chr5:35313365-35313892	*Adra2c/*4930478P22Rik*
chr5:36830677-36831279	*Man2b2/Ppp2r2c*
chr5:37040212-37041347	*Jakmip1/Wfs1*
chr5:52515885-52516367	*Ccdc149/Lgi2*
chr5:105732003-105732105	*Lrrc8d*
chr5:105732186-105732404	*Lrrc8d*
chr5:116082856-116083275	*Tmem233*
chr5:117348264-117349083	Wsb2/Vsig10*
chr5:118268910-118269285	2410131K14Rik*/Med13*
chr5:120761506-120761888	*Oas3*
chr5:120783028-120783454	*Oas3/Oas1e*
chr5:136245578-136246023	*Sh2b2/Cux1*
chr5:137071619-137072273	Serpine1*
chr5:143128699-143129529	*Rnf216/Rbakdn*
chr5:143133061-143133869	*Rnf216/Rbakdn*
**chr6:4746229-4747314**	Sgce*
**chr6:4747440-4747696**	Peg10*
**chr6:4747780-4747996**	Peg10*
**chr6:4748054-4749480**	Peg10*
chr6:30732981-30733591	Mest/Cep41*
**chr6:30735435-30736116**	Mest*
**chr6:30736607-30738780**	Mest*
**chr6:30739031-30740864**	Mest/Mir335*
chr6:34948776-34948940	*Stra8/*2010107G12Rik
chr6:37464011-37464558	*Creb3l2/Akr1d1*
chr6:39269183-39269977	*Kdm7a/Slc37a3*
**chr6:58906186-58907095**	Nap1l5/Herc3*
chr6:85378234-85378818	*Rab11fip5/Noto*
chr6:107531195-107531557	*Lrrn1/Inpp5f*
chr6:115729908-115730603	Tmem40 *
chr6:119594548-119594870	*Wnt5b*
chr6:125349316-125349717	Tnfrsf1a/Scnn1a*
chr6:130973317-130980712	*Klra2/Klra22/Klra15*
chr6:136518731-136518889	*Atf7ip*
chr7:4531968-4532355	*Dnaaf3*
**chr7:6727076-6727722**	Peg3*
**chr7:6727892-6732060**	Peg3*
chr7:10324647-10325420	*Vmn1r66/Vmn1r67*
chr7:16893755-16894739	*Gng8*
chr7:19175647-19176796	Eml2/Gipr*
chr7:19811098-19811297	Bcl3*
chr7:24611394-24611596	*Phldb3*
chr7:25301189-25301458	*Prr19*
chr7:25903349-25903407	Cyp2b10*
chr7:29211649-29211960	*Catsperg1*
chr7:33836946-33837410	*Scgb1b24/Scgb2b26*
chr7:41964212-41974972	*Vmn2r59/Vmn2r58*
chr7:45340239-45340780	*Ppfia3*
**chr7:60002938-60005042**	Snurf*
**chr7:60005123-60008428**	Snurf*
chr7:102289205-102289935	*Stim1*
**chr7:128687633-128688728**	Inpp5f*
chr7:142577780-142578554	H19*
**chr7:142580201-142582623**	H19/IGF2*
**chr7:143293662-143295410**	Kcnq1ot1/Kcnq1*
**chr7:143295635-143297239**	Kcnq1ot1/Kcnq1*
chr8:3656464-3657513	*Retn*
chr8:27222728-27224119	Adrb3/Got1l1*
chr8:56623073-56623637	*Fbxo8/Hand2*
chr8:71687314-71688316	*Insl3/Jak3*
chr8:80917179-80918973	Gab1/Usp38*
chr8:94153505-94154175	Mt3*
chr8:105374715-105374834	*Plekhg4/Slc9a5*
chr8:109075175-109075765	D030068K23Rik
chr8:117109911-117110338	*Bco1*
chr8:121273267-121273661	*Foxl1/*1700018B08Rik
chr8:121541793-121542193	1700018B08Rik
chr8:121542486-121543053	170018B08Rik/30M09Rik
chr8:122864817-122865344	*Cdh15*
chr8:124363052-124363447	Pgbd5/Galnt2*
chr8:126476627-126476857	*Gm17296/Irf2bp2*
chr9:3199699-3199906	4930433N12Rik
chr9:20857639-20859264	A230050P20Rik/Rdh8*
chr9:20911944-20912365	*Dnmt1*
chr9:45115913-45116264	*Scn2b/*Gm10684
chr9:89737795-89738762	Ankrd34c*
**chr9:89879711-89881749**	***Rasgrf1***/4930524008Rik*
chr9:91380349-91381121	*Zic4*
chr10:7614884-7617224	*Lrp11*
**chr10:13090448-13091600**	Plagl1*
chr10:79735189-79735518	Polrmt/Hcn2/Bc1*
chr11:4440809-4440945	*Hormad2*
chr11:5516696-5516994	Xbp1/Znrf3*
**chr11:12025737-12025932**	Grb10*
**chr11:12025971-12026410**	Grb10*
**chr11:12026426-12026880**	Grb10*
chr11:22007227-22007753	Otx1/Ehbp1*
**chr11:22971545-22974429**	Zrsr1/Commd1*
chr11:54807023-54807079	*Lyrm7os/Cdc42se2*
chr11:58961910-58961988	*Trim17*
chr11:67084007-67084273	*Myh3*
chr11:115441364-115441906	Trim80*
chr11:117479477-117479864	Gm11733*/Sept9*
chr11:117790191-117790485	6030468B19Rik*/Tmc8*
chr11:119258377–119259019	*Gaa/Ccdc40*
chr11:120316425-120316754	*Actg1/Bahcc1*
chr11:120949785-120950433	*Slc16a3*
chr11:121519413-121520621	Zfp750/Tbcd*
chr12:71577311-71577941	4930404H11Rik/Daam1*
chr12:84640456-84641612	*Vrtn*
chr12:104448190-104448500	*Gsc/Serpina3n*
**chr12:109524554-109526114**	Meg3/Dlk1*
**chr12:109527558-109529443**	Meg3/Dlk1*
**chr12:109529532-109531222**	Meg3/Dlk1*
chr12:109539200-109539966	Meg3/Dlk1*
chr12:109541027-109541475	Meg3*
chr12:109541501-109542870	Meg3*
chr13:12833830-12837749	*Prl2c3/Prl2c2*
chr13:13450125-13451423	*Nid1*
chr13:23285704-23286065	4933404K08Rik
chr13:23286248-23286415	4933404K08Rik
chr13:23299932-23300187	4933404K08Rik
chr13:23301151-23301633	4933404K08Rik
chr13:23306774-23307322	4933404K08Rik
chr13:23313146-23313412	4930557F10Rik
chr13:30947041-30947819	Hus1b/Exoc2*
chr13:47013723-47014359	*Nhlrc1*
chr13:52928754-52929435	*Auh*
chr13:53194451-53194648	*Ror2*
chr13:56522315-56522451	*Fbxl21/ll9*
chr13:84236742-84237442	*Tmem161b*
chr13:104531878-104532411	*Adamts6/Cwc27*
chr13:120024970-120025495	B020031M17Rik/Gm20767
chr13:120026729-120027576	B020031M17Rik/Gm20767
chr13:120028688-120029477	HB020031M17Rik/Gm20767*
chr13:120030479-120030889	GM20767/B020031M17Rik
chr13:120033528-120033703	Gm21188/B020031M17Rik
chr13:120035672-120036958	Gm21188*
chr14:58073331-58073940	*Fg19*
chr14:65404585-65404937	*Pnoc*
chr14:68122518-68124228	A230070E04Rik/*Nefm*
chr14:68124336-68124471	*Nefm*/ A230070E04Rik
chr14:79772279-79772640	*Pcdh8*/Gm10845
chr15:27476183-27476613	*Ank*
**chr15:72809195-72811003**	Peg13/Trappc9*
chr15:78037646-78038392	*Cacng2*
chr15:79972322-79972965	*Cbx7/Pdgfb*
chr15:85131470-85131874	*Smc1b*
chr16:11035880-11036418	*Snn*/Gm4262
chr16:20732118-20732136	*Chrd/Thpo*
chr16:22405637-22406912	Etv5*
chr16:22407226-22408474	Etv5*
chr16:22410190-22411163	Etv5*
chr17:5958515-5959056	*Synj2*
chr17:6582455-6582783	*Dynlt1c*
**chr17:12741566-12742455**	Airn/Igf2r*
**chr17:12742478-12742859**	Airn/Igf2r*
chr17:21383671-21383999	*Zfp677*
chr17:26603052-26603573	Ergic1*
chr17:28858375-28858617	*Pnpla1*
chr17:30874696-30874978	*Glp1r/Dnah8*
chr17:57492974-57493278	*Vmn2r120/Emr1*
chr17:83874898-83875201	*Haao*/4933433H22Rik
**chr18:12972131-12975659**	Impact*
chr18:24671762-24671989	*Mocos*
chr18:25478389-25478493	*Celf4*
chr18:36940664-36940927	*Pcdha2/Pcdha1*
chr18:36992516-36992804	*Pcdha8/Pcdha1*/Gm37013
chr18:37012737-37013324	*Pcdha11/Pcdha1*/Gm37013
chr18:37402798-37403276	*Pcdhb9*/Gm37013/Gm38666
chr18:65698431-65698803	*Oacyl*
chr19:16035301-16036310	*C130060C02Rik/Gnaq*
chr19:61225200-61225451	*Csf2ra*

* Indicates that NORED region overlaps bimodal (MethylMoscaic) region

**Bold** indicates NORED region overlaps known imprinted gASM
