# Supplementary material for: Two approaches reveal a new paradigm of ‘switchable or genetics-influenced allele-specific DNA methylation’ with potential in human disease
Source: Cell Discov. 2017 Nov 14;3:17038–. doi: 10.1038/celldisc.2017.38 (PMC5787696; doi:10.1038/celldisc.2017.38)
Supplement: Supplementary Information [file celldisc201738-s1.pdf]

Supplementary Information

Supplementary Table1. Methylation across well-characterized, imprinted germline ASMs
Excel File

Supplementary Table 2. List of 2468 NORED regions
Excel File

Supplementary Table 3. List of 2487 MethylMosaic bimodal regions
Excel File

Supplementary Table 4. Gene set enrichment for 2468 NORED regions
Excel File

Supplementary Table 5. Gene set enrichment for 2487 MethylMosaic bimodal regions
Excel File

Supplementary Table 6. Gene set enrichment for 207 bimodal NORED regions
Excel File

Supplementary Table 7. Gene set enrichment for 2261 NORED exclusive regions
Excel File

Supplementary Table 8. Gene set enrichment for 2335 MethylMosaic bimodal exclusive regions
Excel File

Supplementary Figure 1. Coverage and methylation level for WT, 1KO, r1KO, DKO, and TKO ESCs. (a - e) Coverage per CpG site in WT (a), 1KO (b), r1KO (c), DKO (d), and TKO (e) ESCs. (f) Distribution of CpG methylation for CpG sites with minimum coverage of five in ESCs. Global average methylation for all covered CpG sites in WT, 1KO, r1KO, DKO, and TKO was 0.727, 0.176, 0.369, 0.157, 0.006, respectively.

Supplementary Figure 2. NORED method evaluation. (a) ROC-like curve for individual CpG sites that meet three NORED criteria at various cutoffs for WT, 1KO, and r1KO. Cutoff for WT parameter is a circle for 0.25 and a triangle for 0.30. Cutoff for 1KO parameter is indicated by color. Each circle/triangle represents a tested r1KO value for combinations of WT and 1KO. For r1KO values are between WT and 1KO cutoffs at increments of 0.005. Circle with black outline indicates values for three parameters to select individual CpG sites to combine into regions (r1KO = 0.125). (b) Proportion of gASMs identified compared to region-level FDR

Supplementary Figure 3. MethylMosaic method evaluation. (a) ROC-like curve for CpG-centered windows at various cutoffs for hypermethylation and hypomethylation indices (see Methods) Red point indicates bounds for indices (between 0.2 to 0.75) to select CpG-centered windows to combine into regions. (b) Proportion of gASMs identified at region-level FDR.

Supplementary Figure 4. Overlap of NORED and MethylMosaic demonstrates that two methods are independent, but complementary. (a) Proportion of NORED regions that are also bimodal in MethylMosaic at various FDRs for each method. Total number of NORED regions at each FDR cutoff is listed to the right. (b) Proportion of MethylMosaic regions that are also NORED at various FDRs for each method. Total number of MethylMosaic regions at each FDR cutoff is listed below.

Supplementary Figure 5. Genomic sequencing confirms that WT ESCs are homozygous at *Hcn2/Polrmt* locus.

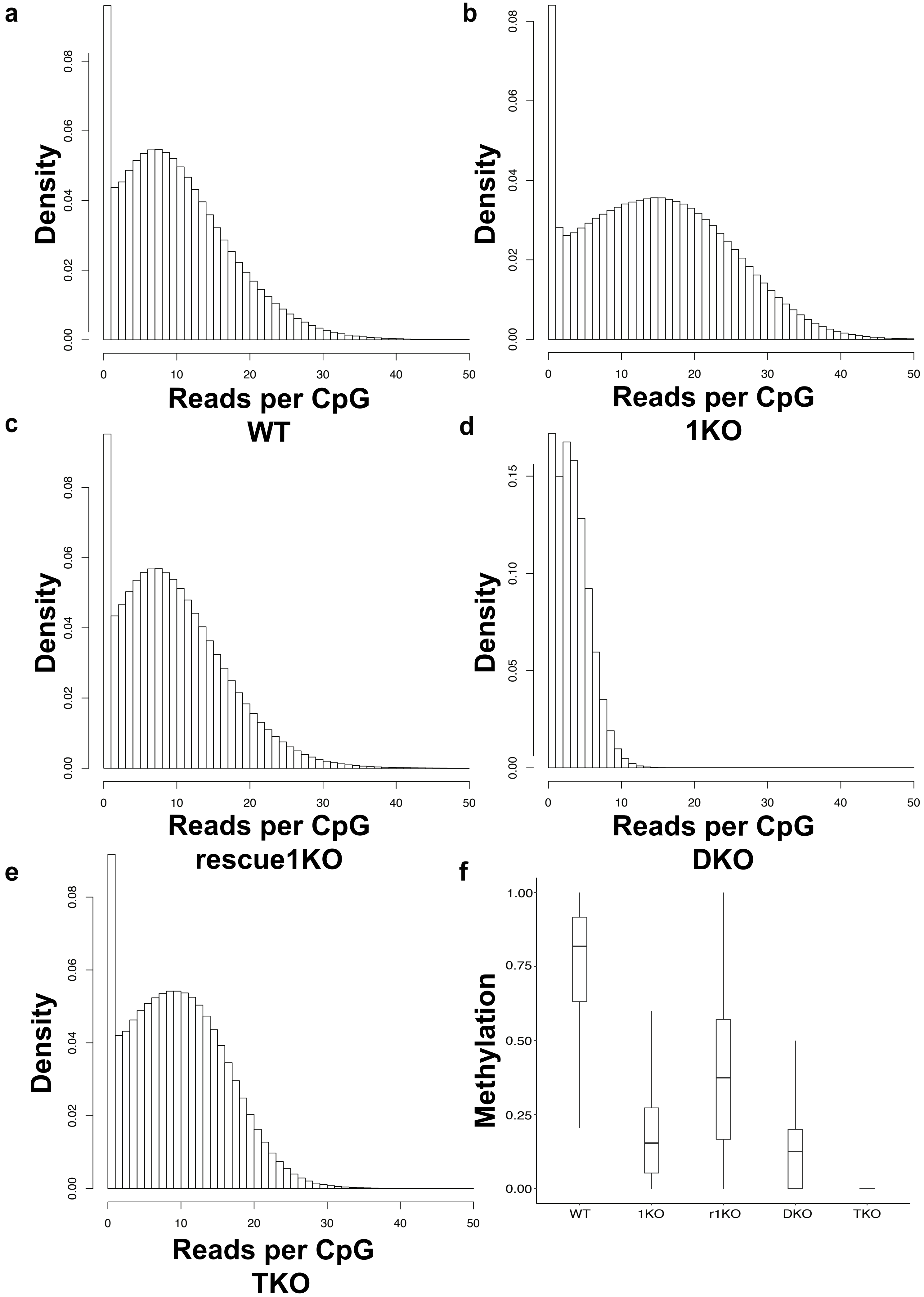

a
Density
Reads per CpG
WT
b
Density
Reads per CpG
1KO
c
Density
Reads per CpG
rescue1KO
d
Density
Reads per CpG
DKO
e
Density
Reads per CpG
TKO
f
Methylation
WT
1KO
r1KO
DKO
TKO

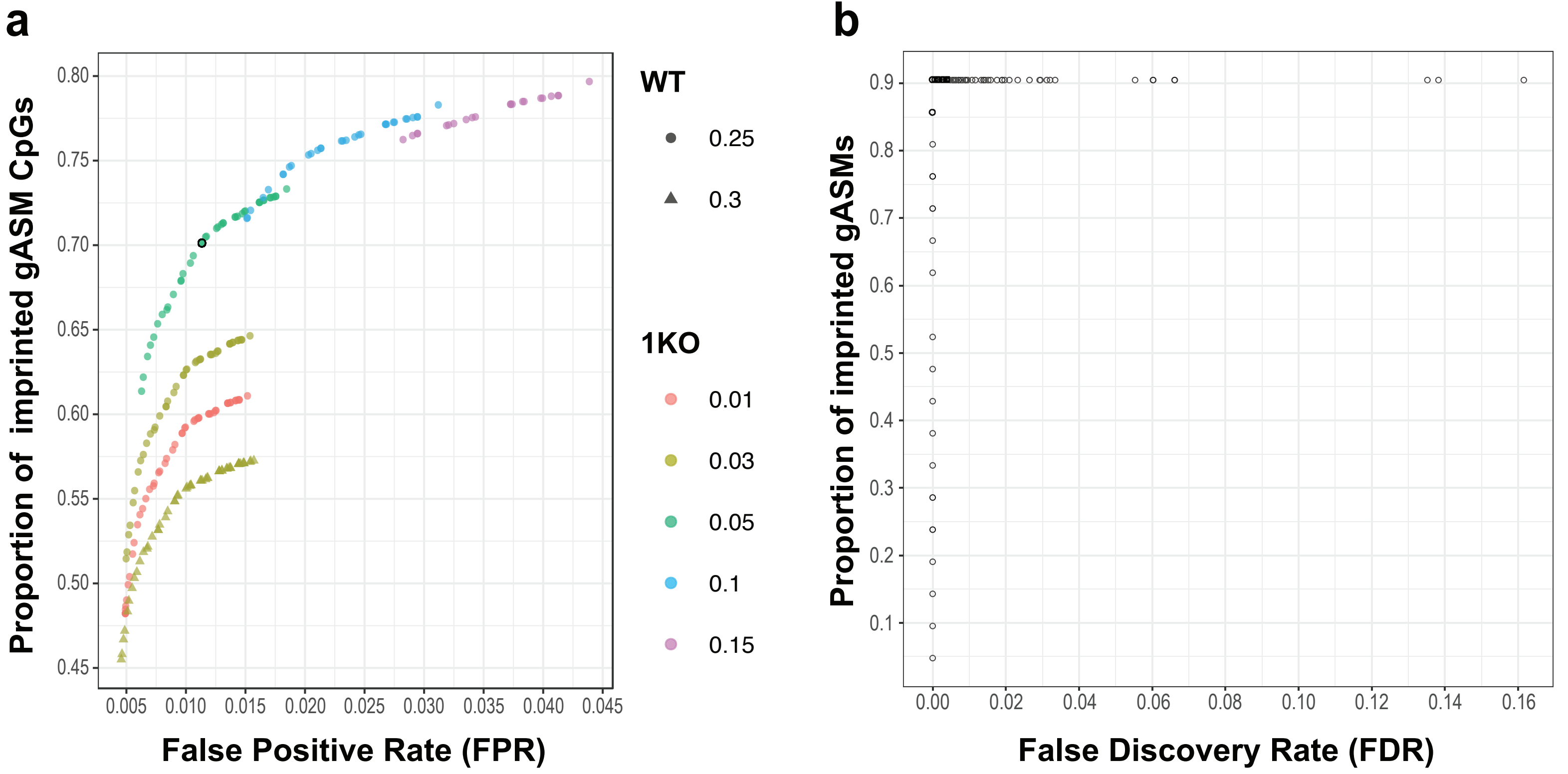

a
Proportion of imprinted gASM CpGs
0.80
0.75
0.70
0.65
0.60
0.55
0.50
0.45
0.005
0.010
0.015
0.020
0.025
0.030
0.035
0.040
0.045
False Positive Rate (FPR)
WT
0.25
0.3
1KO
0.01
0.03
0.05
0.1
0.15
b
Proportion of imprinted gASMs
0.9
0.8
0.7
0.6
0.5
0.4
0.3
0.2
0.1
0.00
0.02
0.04
0.06
0.08
0.10
0.12
0.14
0.16
False Discovery Rate (FDR)

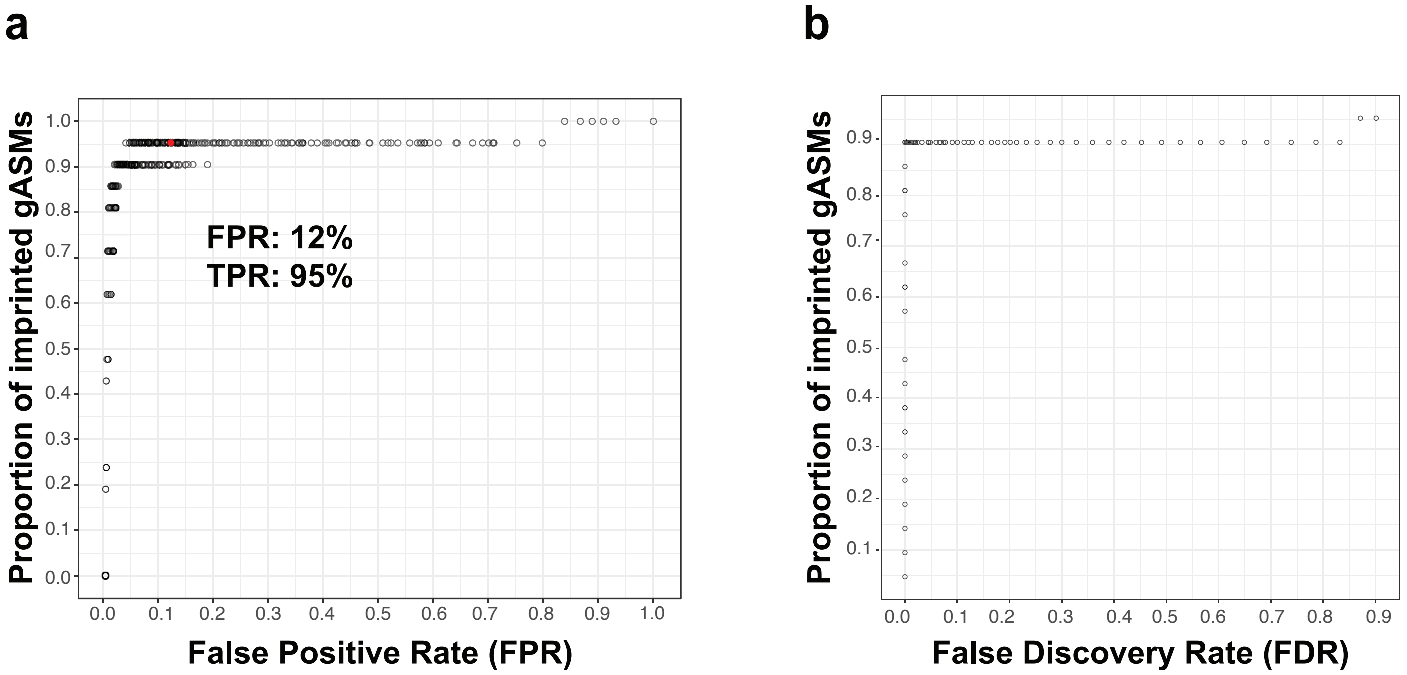
a
FPR: 12%
TPR: 95%
Proportion of imprinted gASMs
False Positive Rate (FPR)
b
Proportion of imprinted gASMs
False Discovery Rate (FDR)

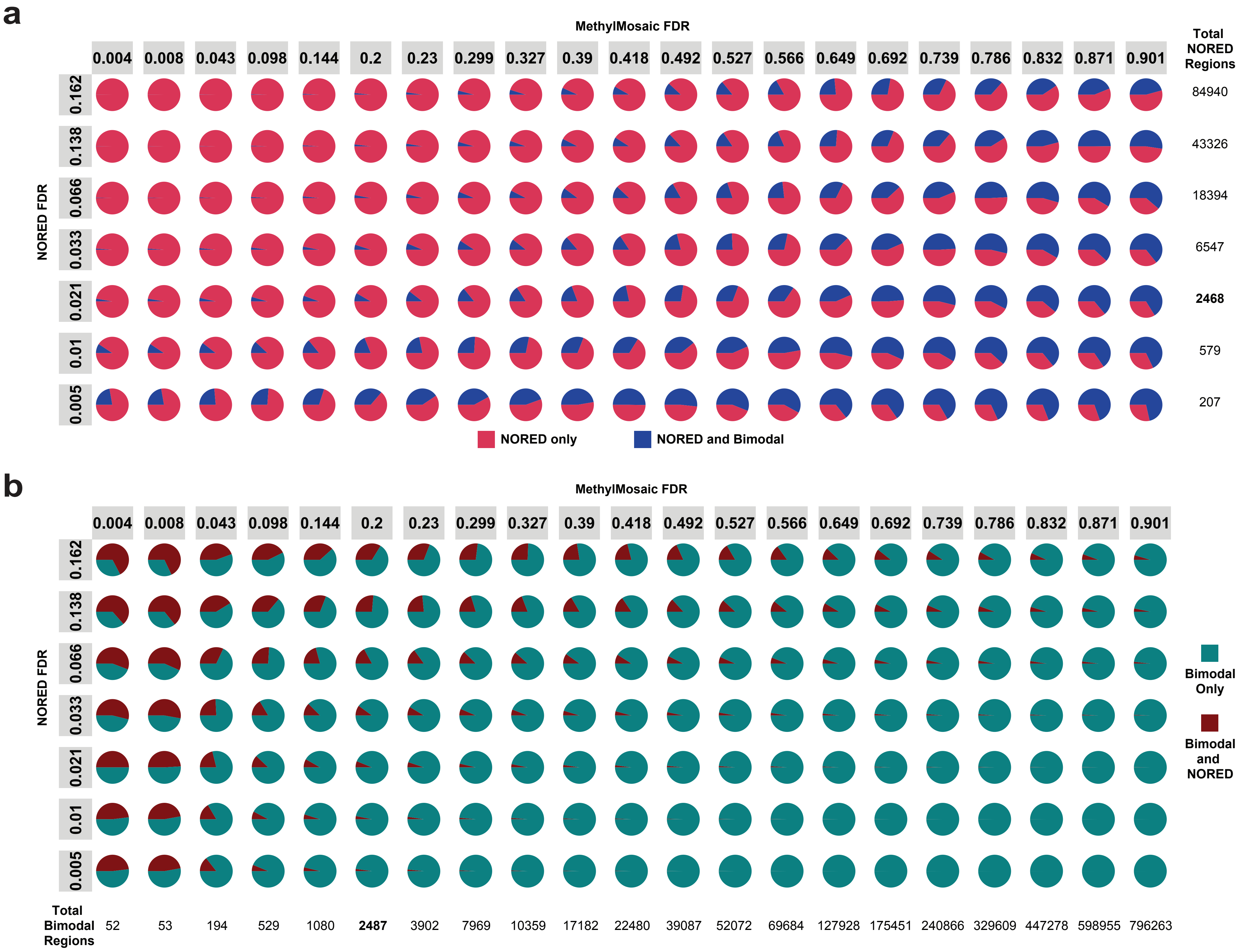

a
MethylMosaic FDR
0.004
0.008
0.043
0.098
0.144
0.2
0.23
0.299
0.327
0.39
0.418
0.492
0.527
0.566
0.649
0.692
0.739
0.786
0.832
0.871
0.901
Total NORED Regions
NORED FDR
0.162
0.138
0.066
0.033
0.021
0.01
0.005
84940
43326
18394
6547
2468
579
207
NORED only
NORED and Bimodal
b
MethylMosaic FDR
0.004
0.008
0.043
0.098
0.144
0.2
0.23
0.299
0.327
0.39
0.418
0.492
0.527
0.566
0.649
0.692
0.739
0.786
0.832
0.871
0.901
NORED FDR
0.162
0.138
0.066
0.033
0.021
0.01
0.005
Bimodal Only
Bimodal and NORED
Total Bimodal Regions
52
53
194
529
1080
2487
3902
7969
10359
17182
22480
39087
52072
69684
127928
175451
240866
329609
447278
598955
796263

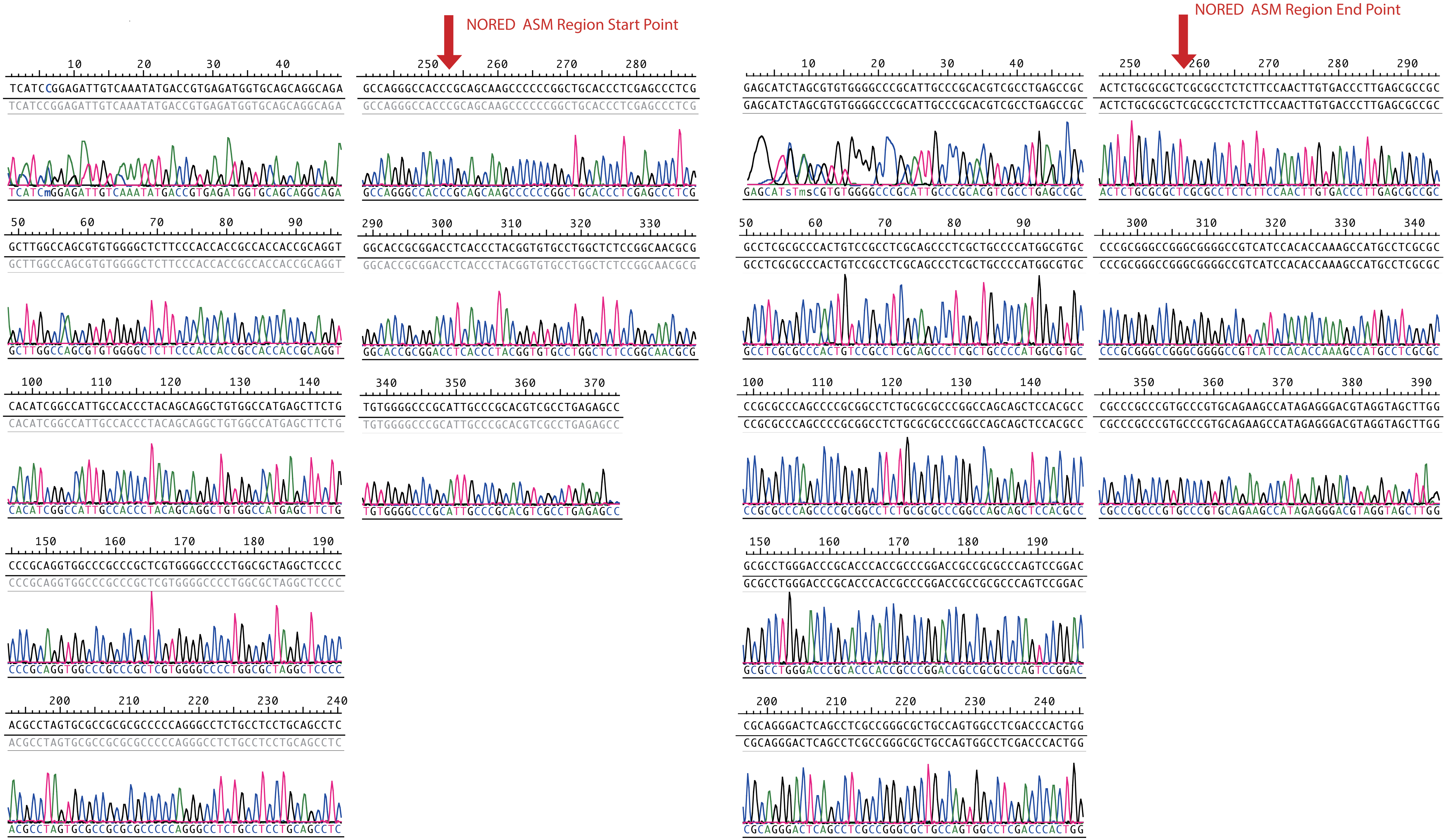

NORED ASM Region Start Point
NORED ASM Region End Point
